# Evidence for causal effects of lifetime smoking on risk for depression and schizophrenia: a Mendelian randomisation study

**DOI:** 10.1017/S0033291719002678

**Published:** 2020-10

**Authors:** Robyn E. Wootton, Rebecca C. Richmond, Bobby G. Stuijfzand, Rebecca B. Lawn, Hannah M. Sallis, Gemma M. J. Taylor, Gibran Hemani, Hannah J. Jones, Stanley Zammit, George Davey Smith, Marcus R. Munafò

**Affiliations:** 1 School of Experimental Psychology, University of Bristol, Bristol BS8 1TU, UK; 2 MRC Integrative Epidemiology Unit, University of Bristol, Bristol BS8 2PR, UK; 3 NIHR Biomedical Research Centre at the University Hospitals Bristol NHS Foundation Trust and the University of Bristol, Bristol BS8 2BN, UK; 4 Department of Population Health Sciences, Bristol Medical School, University of Bristol, Bristol BS8 2PR, UK; 5 Jean Golding Institute, Royal Fort House, University of Bristol, Bristol BS8 1UH, UK; 6 Department of Psychology, Addiction and Mental Health Group (AIM), University of Bath, Bath BA2 7AY, UK; 7 Division of Psychological Medicine and Clinical Neurosciences, MRC Centre for Neuropsychiatric Genetics and Genomics, Cardiff University, Cardiff CF24 4HQ, UK; 8 UK Centre for Tobacco and Alcohol Studies, University of Bristol, Bristol BS8 1TU, UK

**Keywords:** ALSPAC, depression, Mendelian randomisation, schizophrenia, smoking, tobacco, UK Biobank

## Abstract

**Background:**

Smoking prevalence is higher amongst individuals with schizophrenia and depression compared with the general population. Mendelian randomisation (MR) can examine whether this association is causal using genetic variants identified in genome-wide association studies (GWAS).

**Methods:**

We conducted two-sample MR to explore the bi-directional effects of smoking on schizophrenia and depression. For smoking behaviour, we used (1) smoking initiation GWAS from the GSCAN consortium and (2) we conducted our own GWAS of lifetime smoking behaviour (which captures smoking duration, heaviness and cessation) in a sample of 462690 individuals from the UK Biobank. We validated this instrument using positive control outcomes (e.g. lung cancer). For schizophrenia and depression we used GWAS from the PGC consortium.

**Results:**

There was strong evidence to suggest smoking is a risk factor for both schizophrenia (odds ratio (OR) 2.27, 95% confidence interval (CI) 1.67–3.08, *p* < 0.001) and depression (OR 1.99, 95% CI 1.71–2.32, *p* < 0.001). Results were consistent across both lifetime smoking and smoking initiation. We found some evidence that genetic liability to depression increases smoking (*β* = 0.091, 95% CI 0.027–0.155, *p* = 0.005) but evidence was mixed for schizophrenia (*β* = 0.022, 95% CI 0.005–0.038, *p* = 0.009) with very weak evidence for an effect on smoking initiation.

**Conclusions:**

These findings suggest that the association between smoking, schizophrenia and depression is due, at least in part, to a causal effect of smoking, providing further evidence for the detrimental consequences of smoking on mental health.

## Introduction

Smoking is a major risk factor for lung cancer, cardiovascular and respiratory diseases making it the leading cause of preventable death worldwide (World Health Organization, 2011). In developed nations, smoking is more common amongst individuals with mental health conditions (Coulthard, Farrell, Singleton, & Meltzer, 2002; Lasser et al., 2000; Lawrence, Mitrou, & Zubrick, 2009; McClave, McKnight-Eily, Davis, & Dube, 2010), in particular schizophrenia (Royal College of Physicians, 2013) and depression (Byers et al., 2012; Leung, Gartner, Dobson, Lucke, & Hall, 2011; Tjora et al., 2014). In the UK, estimates suggest that up to 45% of individuals with schizophrenia, and 31% of individuals with depression smoke (Royal College of Physicians, 2013), compared with around 15% of the general population (Office for National Statistics, 2019). Individuals with mental health conditions smoke more heavily (Coulthard et al., 2002) and experience up to 18 years reduced life expectancy compared with the general population (Chang et al., 2011; Royal College of Physicians, 2013). Much of this reduction can be explained by smoking-related diseases (Royal College of Physicians, 2013), making it important to understand the relationship between smoking and mental health.

It is often assumed that the association between mental health and smoking can be explained by a self-medication model – that is, symptoms of mental illness, or side effects of psychiatric medications, are alleviated by the chemical properties of tobacco (Desai, Seabolt, & Jann, 2001; Khantzian, 1997; Lerman et al., 1996; Levin, Wilson, Rose, & McEvoy, 1996). However, observational evidence cannot determine whether the association between smoking and mental health is causal or the result of confounding (Lawlor, Harbord, Sterne, Timpson, & Davey Smith, 2008). Furthermore, traditional observational evidence cannot robustly identify the direction of causation (Lawlor et al., 2008), and there is growing evidence to suggest that smoking may be a causal risk factor for poor mental health. The genome-wide association study (GWAS) of schizophrenia conducted by the Psychiatric Genomics Consortium (PGC) found that variants in the gene cluster *CHRNA5-A3-B4* were associated with increased schizophrenia risk (Ripke et al., 2014). These variants are known to be strongly associated with heaviness of smoking (Munafò et al., 2012; Thorgeirsson et al., 2008; Tobacco Consortium, 2010; Ware, van den Bree, & Munafò, 2011). Therefore, one interpretation is a possible causal effect of smoking on schizophrenia (Gage & Munafò, 2015). Furthermore, there is evidence of genetic correlations between smoking, schizophrenia and depression (Hartz et al., 2018; Liu et al., 2019) warranting further investigation of possible causal effects. Prospective observational studies using related individuals to control for genetic and environmental confounding have suggested a dose–response effect of smoking on schizophrenia (Kendler, Lönn, Sundquist, & Sundquist, 2015) and depression (Kendler et al., 1993). Meta-analyses show further evidence for an increased relative risk of schizophrenia in smokers over non-smokers (Gurillo, Jauhar, Murray, & MacCabe, 2015; Scott et al., 2018) and a reduction in depressive symptoms following smoking cessation (Taylor, McNeill et al., 2014). Although these studies suggest a potential causal effect, more robust methods are required to triangulate evidence and allow for stronger causal inference (Munafò & Davey Smith, 2018).

One possible way to overcome bias from residual confounding and reverse causation is Mendelian randomisation (MR) (Davey Smith & Ebrahim, 2003). This method uses genetic variants to proxy for an exposure in an instrumental variable analysis to estimate the causal effect on an outcome (Lawlor et al., 2008). Previous MR studies have failed to show any clear evidence for an effect of smoking on depression (Bjørngaard et al., 2013; Taylor, Fluharty et al., 2014; Wium-Andersen, Ørsted, & Nordestgaard, 2015) and show suggestive but inconclusive evidence for an effect of smoking on schizophrenia (Gage et al., 2017; Wium-Andersen et al., 2015). However, the genetic instruments for smoking used in these MR studies are limited, only capturing individual aspects of smoking behaviour and only having identified limited numbers of suitable genetic variants (Bjørngaard et al., 2013; Gage et al., 2017; Taylor, Fluharty et al., 2014; Wium-Andersen et al., 2015). Furthermore, any instrument for smoking heaviness requires stratifying samples into smokers and non-smokers. Stratification is not possible using the most common MR method, two-sample MR. In two-sample MR, single-nucleotide polymorphism (SNP)-exposure and SNP-outcome effects are estimated in two independent samples and the effect sizes are obtained from GWAS summary statistics. Therefore, in this context stratification is not possible because GWAS summary data do not provide individual level data regarding smoking status.

Here we improve upon previous MR studies of smoking and mental health in two ways: (1) by using an updated instrument for smoking initiation which comprises 378 SNPs (Liu et al., 2019) and (2) we develop a novel genetic instrument for lifetime smoking exposure that takes into account smoking status (i.e. ever and never smokers), and among ever smokers takes into account smoking duration, heaviness and cessation. This instrument can therefore capture smoking behaviours beyond initiation and can be used in unstratified samples of smokers and non-smokers. By using the results of these different MR analyses together and triangulating across multiple methods, we aim to determine whether the observational associations between smoking, schizophrenia and depression are likely causal, and the directionality of these relationships.

## Methods

### Data sources and genetic instruments

#### Smoking initiation

The most recent GWAS of smoking initiation from the GSCAN consortium identified 378 conditionally independent genome-wide significant SNPs in a sample of 1 232 091 individuals of European ancestry (Liu et al., 2019). The 378 SNPs explain 2% of the variance in smoking initiation (Liu et al., 2019). When smoking initiation was the outcome, summary statistics without 23andMe (*N* = 599 289) were used.

#### Lifetime smoking

To capture smoking heaviness and duration as well as smoking initiation, we generated a lifetime smoking measure using data from the UK Biobank. The UK Biobank is a national health research resource of 502 647 participants aged 40–69 years, recruited from across the UK between 2006 and 2010 (http://www.ukbiobank.ac.uk). Our sample consisted of 462 690 individuals of European ancestry who had phenotype data and passed genotype inclusion criteria (54% female; mean age = 56.7 years; s.d. = 8.0 years). Overall, 30% of the sample had ever smoked (8% current smokers and 22% former smokers).

*Measures of smoking*. Smoking measures available in the UK Biobank were self-reported and collected at initial assessment. They included: smoking status (current, former, never – field 20116), age at initiation in years (fields 3436/2867), age at cessation in years (field 2897) and number of cigarettes smoked per day (fields 3456/2887). Anyone self-reporting to smoke more than 100 cigarettes per day was contacted for confirmation. Hand-rolled cigarette smokers were told 1 g of tobacco equates to one cigarette. We calculated duration of smoking and time since cessation. UK Biobank removed individuals smoking fewer than 1 or more than 150 cigarettes a day.

*Construction of the lifetime smoking index*. Following the method outlined by Leffondré, Abrahamowicz, Xiao, and Siemiatycki (2006), we combined the smoking measures into a lifetime smoking index along with a simulated half-life (*τ*) constant. Half-life captures the exponentially decreasing effect of smoking at a given time on health outcomes. The value of half-life was determined by simulating the effects of lifetime smoking on lung cancer and overall mortality in the UK Biobank. Both suggested the best fitting value as 18. For full details on construction of the lifetime smoking index see online Supplementary Note.

*Genome-wide association study of lifetime smoking index*. For full details of genotyping and exclusion procedures see online Supplementary Note. After excluding individuals who did not pass genotype exclusions and who had missing phenotype data, 462 690 individuals remained for the GWAS. Of these individuals, 249 318 were never smokers (54%), 164 649 were former smokers (36%) and 48 723 (11%) were current smokers. The mean value of lifetime smoking score was 0.359 (s.d. = 0.694). GWAS was conducted using the UK Biobank GWAS pipeline set-up for the MRC Integrative Epidemiology Unit (Elsworth et al., 2017). BOLT-LMM was used to conduct the analysis (Loh et al., 2015), which accounts for population stratification and relatedness using linear mixed modelling. Genotyping chip and sex were included as covariates. As a sensitivity analysis, we reran the GWAS without controlling for genotype chip because the UK BiLEVE sub-sample (which used a different genotyping chip) was selected on the basis of smoking status. Genome-wide significant SNPs were selected at *p* < 5 × 10^−8^ and were clumped to ensure independence at linkage disequilibrium (LD) *r*^2^ = 0.001 and a distance of 10 000 kb using the TwoSampleMR package (Hemani et al., 2018).

**Fig. 1.**
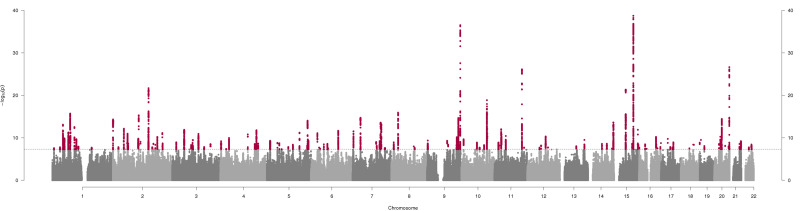
Manhattan plot of genome-wide association study of lifetime smoking index (*N* = 462 690). The *x*-axis represents chromosomal position and the *y*-axis represents −log_10_ *p* value for the association of each SNP with the lifetime smoking index using an additive model and linear regression. The dashed line indicates the genome-wide level of significance (*p* < 5 × 10^−8^) and genome-wide significant SNPs are indicated in red.

*Instrument generated*. Our GWAS of lifetime smoking identified 126 independent, genome-wide significant SNPs (see Fig. 1 and online Supplementary Table S1). A standard deviation increase in the lifetime smoking score is equivalent to an individual smoking 20 cigarettes a day for 15 years and stopping 17 years ago or an individual smoking 60 cigarettes a day for 13 years and stopping 22 years ago. We validated our instrument in an independent sample and using positive control outcomes of lung cancer, coronary heart disease and hypomethylation at the aryl-hydrocarbon receptor repressor site cg05575921 because smoking has been robustly shown to predict these outcomes (WHO, 2011; Zeilinger et al., 2013). For further details of these validations see the online Supplementary Note.

#### Schizophrenia

We used summary data from the PGC consortium GWAS, which comprises 36 989 cases and 113 075 controls of European and East Asian ancestry (Ripke et al., 2014). Cases were a combination of individuals with schizophrenia and schizoaffective disorder, mostly diagnosed by clinicians, but some samples used research-based assessment. The ascertainment method did not affect GWAS results (Ripke et al., 2014). A sensitivity analysis was performed using the GWAS summary data meta-analysed with a further 11 260 cases and 24 542 controls (Pardiñas et al., 2018). The genetic instrument for schizophrenia came from the PGC GWAS, which identified 114 independent SNPs at 108 loci explaining around 3.4% of the variance in schizophrenia liability (Ripke et al., 2014).

#### Depression

For depression, we used GWAS summary data from the PGC for major depression, which comprises 130 664 major depression cases and 330 470 controls of European ancestry (Wray et al., 2018). Cases were either diagnosed with major depressive disorder (MDD) on inpatient or medical health records or self-reported having a diagnosis or treatment for depression. Therefore, the authors use the term major depression over diagnosed MDD (Wray et al., 2018). 23andMe data (75 607 cases and 231 747 controls) were excluded when major depression was the outcome because genome-wide summary statistics are not available with 23andMe data included. The genetic instrument for major depression from the PGC GWAS was 40 genome-wide significant SNPs which explain 1.9% of the variance in liability (Wray et al., 2018).

### Statistical analysis

MR analyses were run using the MR Base R package (Hemani et al., 2018; R. Core Team, 2014) and compared across five different methods: inverse-variance weighted, MR Egger (Bowden, Davey Smith, & Burgess, 2015), weighted median (Bowden, Davey Smith, Haycock, & Burgess, 2016), weighted mode (Hartwig, Smith, & Bowden, 2017) and MR RAPS (Zhao, Wang, Hemani, Bowden, & Small, 2018). Each of these methods makes slightly different assumptions about the nature of pleiotropy and therefore a roughly consistent point estimate across the multiple methods provides the strongest evidence of causal inference (Lawlor, Tilling, & Davey Smith, 2016), with the Inverse variance weighted (IVW) method being the main analysis and each other method providing a sensitivity analysis. For additional details on each of the methods see online Supplementary Table S12. Two-sample MR analysis was run bi-directionally, first with smoking as the exposure and then as the outcome. To test the suitability of the MR Egger method, the *I*^2^_GX_ statistic was calculated to quantify the degree of regression dilution bias due to measurement error of SNP-exposure effects (Bowden et al., 2016). We also calculated the mean *F* statistic as an indicator of instrument strength. Steiger filtering was conducted to confirm the direction of effect (Hemani, Tilling, & Davey Smith, 2017). If a SNP from the instrument was unavailable in the outcome, an attempt to find proxies was made with a minimum LD *r*^2^ = 0.8 and palindromic SNPs were aligned with Minor allele frequency < 0.3.

## Results

Bi-directional MR analyses provided strong evidence that higher lifetime smoking increases risk of both schizophrenia [IVW: odds ratio (OR) 2.27, 95% confidence interval (CI) 1.67–3.08, *p* < 0.001] and depression (IVW: OR 1.99, 95% CI 1.71–2.32, *p* < 0.001) (see Table 1), with consistent direction of effect across all five MR methods. The same was seen for smoking initiation as the instrument on schizophrenia (IVW: OR 1.53, 95% CI 1.35–1.74, *p* < 0.001) and depression (IVW: OR 1.54, 95% CI 1.44–1.64, *p* < 0.001) (see Table 1). MR Egger results are the least reliable due to low *I*^2^_GX_ (see online Supplementary Table S3). There was also evidence of a consistent but smaller effect of higher genetic liability for schizophrenia on lifetime smoking (IVW: *β* = 0.022, 95% CI 0.005–0.038, *p* = 0.009) and of genetic liability for depression on lifetime smoking (IVW: *β* = 0.091, 95% CI 0.027–0.155, *p* = 0.005) (see Table 2). These effects remained for depression on smoking initiation (IVW: *β* = 0.083, 95% CI 0.039–0.127, *p* < 0.001) but became even weaker and inconsistent across the different methods for schizophrenia on smoking initiation (IVW: *β* = 0.010, 95% CI 0.000–0.021, *p* = 0.04) (see Table 2). Overall, the effect was larger and more consistent for depression than for schizophrenia. We saw similar effects using the more recent meta-analysed GWAS for schizophrenia with an additional 11 260 cases (see online Supplementary Table S9). There was evidence of significant heterogeneity (see online Supplementary Table S4) but MR Egger intercepts suggest directional pleiotropy is not biasing the estimates (see online Supplementary Table S5) and Steiger filtering supported the conclusion that the effects operate in both directions (see online Supplementary Table S6). Given that variants from the *CHRNA5-A3-B4* gene complex were identified in both GWAS of lifetime smoking and schizophrenia, we conducted a sensitivity analysis removing these variants and the effects remained consistent (see online Supplementary Table S10).

**Table 1.** Two-sample MR analyses of the effect of smoking exposure on schizophrenia and depression

Exposure	Outcome	Method	*N* SNP	OR (95% CI)	*p* value
Lifetime smoking	Schizophrenia	Inverse-variance weighted	125	2.27 (1.67–3.08)	1.36 × 10^−07^
		MR Egger (SIMEX)	125	4.59 (1.49–14.11)	0.009
		Weighted median	125	2.04 (1.57–2.64)	8.21 × 10^−08^
		Weighted mode	125	1.71 (0.69–4.23)	0.25
		MR RAPS	125	2.44 (1.84–3.25)	9.30 × 10^−10^
Lifetime smoking	Depression	Inverse-variance weighted	126	1.99 (1.71–2.32)	9.69 × 10^−19^
		MR Egger (SIMEX)	126	1.09 (0.62–1.92)	0.77
		Weighted median	126	1.97 (1.65–2.35)	3.00 × 10^−14^
		Weighted mode	126	1.83 (1.19–2.81)	0.007
		MR RAPS	126	1.99 (1.70–2.32)	2.76 × 10^−18^
Smoking initiation	Schizophrenia	Inverse-variance weighted	371	1.53 (1.35–1.74)	3.70 × 10^−11^
		MR Egger (SIMEX)	371	1.35 (0.83–2.22)	0.23
		Weighted median	371	1.38 (1.23–1.55)	3.44 × 10^−08^
		Weighted mode	371	1.23 (0.74–2.06)	0.42
		MR RAPS	371	1.63 (1.45–1.85)	4.46 × 10^−15^
Smoking initiation	Depression	Inverse-variance weighted	370	1.54 (1.44–1.64)	3.61 × 10^−37^
		MR Egger (SIMEX)	370	1.36 (1.10–1.67)	0.004
		Weighted median	370	1.46 (1.35–1.58)	4.62 × 10^−21^
		Weighted mode	370	1.44 (1.10–1.89)	0.008
		MR RAPS	370	1.54 (1.44–1.65)	1.31 × 10^−35^

SIMEX, simulation extrapolation, MR RAPS, robust adjusted profile score.

*Note*: Due to low regression dilution *I*^2^_GX_ for lifetime smoking and smoking initiation (see online Supplementary Table S3), MR Egger SIMEX estimates should be interpreted with caution. SIMEX-corrected estimates are unweighted.

**Table 2.** Two-sample MR analyses of the effect of schizophrenia and depression on smoking

Exposure	Outcome	Method	*N* SNP	*β* (95% CI)	*p* value
Schizophrenia	Lifetime smoking	Inverse-variance weighted	102	0.022 (0.005–0.038)	0.009
		MR Egger (SIMEX)	–	–	–
		Weighted median	102	0.015 (0.004–0.026)	0.009
		Weighted Mode	102	0.016 (−0.014 to 0.045)	0.31
		MR RAPS	102	0.018 (0.003–0.032)	0.015
Depression	Lifetime smoking	Inverse-variance weighted	34	0.091 (0.027–0.155)	0.005
		MR Egger (SIMEX)	–	–	–
		Weighted median	34	0.100 (0.058–0.141)	2.77 × 10^−06^
		Weighted mode	34	0.109 (0.037–0.182)	0.005
		MR RAPS	34	0.078 (0.014–0.141)	0.016
Schizophrenia	Smoking initiation	Inverse-variance weighted	107	0.010 (0.000–0.021)	0.04
		MR Egger (SIMEX)	107	−0.030 (−0.093 to 0.033)	0.35
		Weighted median	107	0.003 (−0.006 to 0.012)	0.53
		Weighted Mode	107	−0.008 (−0.033 to 0.017)	0.54
		MR RAPS	107	0.008 (−0.002 to 0.017)	0.11
Depression	Smoking initiation	Inverse-variance weighted	34	0.083 (0.039–0.127)	2.32 × 10^−04^
		MR Egger (SIMEX)	–	–	–
		Weighted median	34	0.077 (0.042–0.112)	1.55 × 10^−05^
		Weighted mode	34	0.062 (0.007–0.117)	0.03
		MR RAPS	34	0.077 (0. 037–0.117)	1.64 × 10^−04^

SIMEX, simulation extrapolation, MR RAPS, robust adjusted profile score.

*Note*: Due to low regression dilution *I*^2^_GX_ (see online Supplementary Table S3), MR Egger estimates could not be conducted apart from for the effect of schizophrenia on smoking initiation, where a weighted MR Egger SIMEX was conducted. Smoking initiation scores are given in *β*s by GSCAN calculated from the meta-analysed *z*-score statistic by assuming a prevalence for the binary trait (Liu et al., 2019).

A total of 14 260 cases and 15 480 controls from the UK Biobank were included in the latest GWAS for major depression (Wray et al., 2018) which could lead to bias from sample overlap (Burgess et al., 2015). Therefore, this analysis was repeated using summary data from an earlier GWAS of major depression (Ripke et al., 2013), which showed a consistent direction of effect with weaker statistical evidence, possibly due to reduced sample size (*N* = 18 759) (see online Supplementary Table S7). Second, we repeated this analysis with the recent meta-analysed GWAS which includes broader definitions of depression including self-declared (Howard et al., 2019). These summary data were available with UK Biobank and 23andMe removed leaving a sample size of 500 199 (170 756 cases and 329 443 controls). The results of this analysis are presented in online Supplementary Table S11. They are relatively consistent with the main analysis, showing slightly larger effects of depression on smoking and slightly reduced effects of smoking on depression. Bi-directional MR analyses were repeated using the GWAS of lifetime smoking without controlling for genotyping chip and the same pattern of results was observed (see online Supplementary Table S8). Further sensitivity tests were conducted and are presented in online Supplementary Figs S9–S20.

## Discussion

We conducted a GWAS of lifetime smoking exposure which provides a novel genetic instrument that can be used in two-sample MR of summary data without the need to stratify on smoking status. We used this novel genetic instrument along with a new instrument of smoking initiation to explore possible causal pathways between smoking, schizophrenia and depression. The two-sample MR results provide strong evidence that smoking is a risk factor for both schizophrenia and depression. This supports prospective observational evidence controlling for genetic confounding (Kendler et al., 1993, 2015), as well as meta-analyses of observational studies (Gurillo et al., 2015; Taylor, McNeill et al., 2014) (although it should be noted that these meta-analyses include estimates not adjusted for known confounders e.g. cannabis use). Effect sizes were similar to a more recent meta-analysis that did adjust for multiple confounders and found a two-fold increased risk of schizophrenia in smokers compared with non-smokers (Scott et al., 2018). Some studies which adjust for potential confounders find the effect of smoking attenuates to the null (Jones et al., 2018) or even becomes protective (Zammit et al., 2003), demonstrating that there are likely to be substantial confounding effects in observational studies. Previous MR studies have not found clear evidence to support smoking as a risk factor for either schizophrenia or depression (Bjørngaard et al., 2013; Gage et al., 2017; Taylor, Fluharty et al., 2014; Wium-Andersen et al., 2015), but our approach offers greater power, captures multiple aspects of smoking behaviour and enables two-sample MR analysis using summary data in unstratified samples. However, it is not possible to precisely estimate from our results what proportion of the observational association between smoking, schizophrenia and depression is causal, or the population attributional fraction of these disorders due to smoking.

In support of the self-medication hypothesis (Khantzian, 1997), we found evidence to suggest that genetic liability for schizophrenia and depression increases lifetime smoking. This supports previous observational evidence (Desai et al., 2001; Lerman et al., 1996; Levin et al., 1996) and might explain why smoking rates remain so high amongst individuals with schizophrenia and depression compared with the general population (Cook et al., 2014). However, evidence was stronger for self-medication effects in depression than schizophrenia and when using smoking initiation as the outcome rather than lifetime smoking, effects attenuated to the null. Therefore, maybe any self-medication effects of schizophrenia are only on heaviness and duration of smoking (captured by the lifetime smoking index) rather than initiation. However, it is important to note that the effects might be weaker because MR methods typically capture the long-term effects of exposures (Labrecque & Swanson, 2018) with self-medication potentially being more acute.

Alternatively the effects of schizophrenia and depression on lifetime smoking might be explained by the misattribution hypothesis. This proposes that smokers misattribute the ability of cigarettes to relieve withdrawal, to their ability to relieve symptoms of psychological distress (Parrott, 1999, 2003). For example, withdrawal symptoms include depressed mood, anxiety and irritability, and smoking a cigarette alleviates those symptoms (Hughes, 2007). Since many withdrawal symptoms are similar to the negative symptoms of schizophrenia and mood symptoms in depression, their alleviation by smoking could give rise to the strong belief that smoking helps to alleviate mental health symptoms.

A potential biological mechanism for the bi-directional causal effects of smoking, schizophrenia and depression could be neuroadaptations in the dopaminergic and serotonin systems. Nicotine acts on nicotinic cholinergic receptors in the brain stimulating the release of neurotransmitters including dopamine and serotonin (Benowitz, 2010). Dopamine and serotonin dysfunction have been implicated in the aetiology of schizophrenia and depression, respectively (Howes, McCutcheon, & Stone, 2015; Jakubovski, Varigonda, Freemantle, Taylor, & Bloch, 2015). It is plausible, therefore, that disruption of these pathways has a causal effect on these disorders. Alternatively, cannabis use could be a mediating mechanism for the effects of smoking on schizophrenia and depression. In prospective studies, cigarette smoking has been shown to increase risk of cannabis dependence (Hindocha et al., 2015). There is strong evidence suggesting that cannabis use increases the risk of psychosis and affective disorders (Moore et al., 2007). This vertical pleiotropy does not violate the assumptions of MR, but simply means there are intermediate mechanisms operating between the exposure and the outcome. However, the strong effects we observe for lifetime smoking suggest that any mediating influence of cannabis use is likely to only partially account for these effects, given the relatively low prevalence of cannabis use [e.g. annual prevalence in the UK of ~7% in 2010 (United Nations Office on Drugs and Crime (UNODC), 2011)]. Multivariable MR analysis of tobacco and cannabis use would help resolve this question.

Finally, although we are using the method of MR to provide stronger causal inference, the best evidence of a causal effect comes from many corroborating lines of evidence from study designs with diverse assumptions. Looking at our current results alongside previous literature, we conclude this strengthens evidence for an effect of smoking on increased risk of depression and schizophrenia. Future work should attempt to elucidate the underlying mechanisms with a hope to intervene, inform public health messages or further advance our knowledge on the aetiology of mental illness. In particular, it will be important to consider other constituents of tobacco smoke to determine whether it is exposure to nicotine or some other constituent that increases risk of schizophrenia and depression. This is particularly important in the context of the recent growth in electronic cigarette use.

### Strengths and limitations

Our study is the first to generate a genetic instrument for lifetime smoking behaviour in a large sample, which allows the use of two-sample MR with summary data from unstratified samples to answer questions about the association between smoking and other health outcomes. However, there are some limitations which should be noted. First, there is evidence to suggest that even after seemingly controlling for population structure in GWAS of samples as large as the UK Biobank, coincident apparent structure remains (Haworth et al., 2019). This might confound the association between smoking and mental health, increasing the risk of false positives. As independent samples with adequate sample size become available, the influence of structure should be further explored. However, it is reassuring that our instrument predicted lifetime smoking in an independent replication sample, where such issues would not arise in the same manner.

Second, sample overlap in two-sample MR can bias results towards the observational estimate (Burgess et al., 2015). There was some sample overlap between the major depression GWAS (Wray et al., 2018) and the UK Biobank (used to derive the lifetime smoking instrument and included in the smoking initiation GWAS) meaning that the effects could be inflated. Therefore, a sensitivity analysis was conducted using a previous GWAS of major depression (Ripke et al., 2013) which showed the same direction of effect despite lower power. This gives us confidence in the bi-directional effects of smoking and depression, despite sample overlap. This sensitivity analysis also addresses recent concerns over the specificity of the most recent GWAS for major depression (Cai et al., 2019). Comparing self-reported ‘seeking help for depression’ with DSM-diagnosed MDD yielded different results (Cai et al., 2019). However, the earlier GWAS of depression did use Diagnostic and statistical manual diagnosed cases only (Ripke et al., 2013) and showed the same direction of effect despite lower power.

Third, including multiple aspects of smoking behaviour could have introduced more potential for horizontal pleiotropy. The more diffuse the definition of smoking, the more lifestyle factors might be correlated, making it especially important to test for horizontal pleiotropy. We conducted multiple sensitivity analyses (which all make different and largely uncorrelated assumptions), and all demonstrated the same direction of effect. This increases our confidence that the results are not biased by pleiotropy. Furthermore, MR Egger intercepts did not show evidence of directional pleiotropy for schizophrenia or depression. However, further work should still attempt to understand the biological mechanisms underpinning the association to reduce the likelihood of pleiotropic effects.

Fourth, schizophrenia and depression are disorders with an average age of onset around early to mid-adulthood (Office for National Statistics, 2018; World Health Organization, 2018). Our measure of lifetime smoking was generated using participants in the UK Biobank aged over 40 years. Therefore, the causal pathway from smoking to schizophrenia and depression risk might initially seem unclear. However, we were not using participants' smoking behaviour at age 40, but rather retrospective lifetime smoking behaviour from age at initiation. It is plausible that smoking behaviour in earlier life could increase risk of later mental health outcomes and exacerbate symptoms, consequently causing more smokers than non-smokers to seek a diagnosis. Furthermore, we saw consistent effects when using smoking initiation as the exposure. Individuals are more likely to initiate smoking prior to the average age at onset of schizophrenia and depression, with 90% of individuals initiating before 18 years of age (Centers for Disease Control and Prevention, 2018). There has also been recent debate in the field about the interpretation of time varying exposures in MR and one way to minimise bias is to use average SNP effects on phenotype across time, as we have done here with our lifetime smoking instrument (Labrecque & Swanson, 2018). We recommend that future studies wishing to examine the effects of smoking on an outcome use multiple instruments for smoking behaviour with consistent evidence across multiple instruments providing the strongest evidence of a causal effect.

Fifth, there is a high degree of zero inflation in the distribution of our lifetime smoking index scores with 54% of the sample being never smokers and therefore receiving a score of zero. We decided not to transform the variable given the desire to have interpretable effect sizes for MR and we decided not to exclude never smokers because our instrument is designed to be used in two-sample MR without the need to stratify into smokers and non-smokers. Despite the zero inflation, we see similar effects for lifetime smoking and smoking initiation suggesting that this has not impaired the score. Sixth, the lifetime smoking score was simulated using all-cause mortality and lung cancer as outcomes. The pattern of association between smoking and lung cancer risk compared with risk for schizophrenia and depression is likely to be different. However, increased mortality amongst individuals with schizophrenia and depression is in large part due to smoking-related mortality (Royal College of Physicians, 2013). The effects were modelled on all-cause mortality and lung cancer but no difference to the best fitting value of half-life was observed. We hope that using all-cause mortality as an outcome makes the lifetime smoking instrument broadly applicable to exploring multiple outcomes. Furthermore, the same effects are observed using smoking initiation as the exposure, which does not include the simulated variable.

Finally, there is known selection bias in the UK Biobank sample, with participants being more highly educated, less likely to be a smoker and overall healthier than the general UK population (Munafo, Tilling, Taylor, Evans, & Davey Smith, 2017). Of the 9 million individuals contacted, only ~5% consented to take part (Munafo et al., 2017). Due to the lack of representativeness in the UK Biobank sample, prevalence and incidence rates will not reflect underlying population levels and there is potential for collider bias. If both smoking and liability for schizophrenia and depression reduce the likelihood of participating in the UK Biobank, then this would induce a negative correlation between schizophrenia or depression and smoking. That is the opposite of the effects observed, suggesting our estimates may, if anything, be conservative.

## Conclusion

In conclusion, we improved upon previous MR studies of smoking and mental health by using an updated instrument of smoking initiation and by developing a novel genetic instrument of lifetime smoking that can be used in two-sample MR of summary data without stratifying on smoking status or reducing power. In two-sample MR analysis, there was evidence of an effect between smoking and mental health in both directions; however, the however, the effects for depression on smoking were stronger than for schizophrenia on smoking. Strong effects of smoking as a risk factor emphasises the detrimental public health consequences of smoking, especially for mental health, and the need to reduce smoking prevalence not only to reduce the burden of physical illness, but also to reduce the burden of mental illness.

## Supplementary material

For supplementary material accompanying this paper visit https://doi.org/10.1017/S0033291719002678. click here to view supplementary material

## Data

Lifetime smoking GWAS summary data is available for download at https://doi.org/10.5523/bris.10i96zb8gm0j81yz0q6ztei23d.
