# Supplementary material for: Evidence for causal effects of lifetime smoking on risk for depression and schizophrenia: a Mendelian randomisation study
Source: Psychol Med. 2019 Nov 6;50(14):2435–43. doi: 10.1017/S0033291719002678 (PMC7610182; doi:10.1017/S0033291719002678)
Supplement: Supplementary file 1 [file S0033291719002678sup001.docx]

**Supplementary Materials**

| **Supplementary Note** | Page 2 |
| --- | --- |
| *Construction of the lifetime smoking index* | Page 2 |
| *Genotyping and exclusion procedure.*  *Instrument discovery for lifetime smoking index*  *Instrument validation – positive control outcomes*  *Instrument validation – replication in an independent sample* | Page 2  Page 3  Page 3  Page 5 |
| *Genetic correlations* | Page 6 |
|  |  |
| **Figures**  Figure S1. Quantile-quantile plot of lifetime smoking index | Page 7 |
| Figure S2. Scatter plot of IVW and sensitivity analyses of lifetime smoking on coronary artery disease | Page 8 |
| Figure S3. Scatter plot of IVW and sensitivity analyses of lifetime smoking on lung cancer | Page 9 |
| Figure S4. Single SNP analysis of lifetime smoking on coronary artery disease | Page 10 |
| Figure S5. Single SNP analysis of lifetime smoking on lung cancer | Page 11 |
| Figure S6. Leave-one-out analysis of lifetime smoking on coronary artery disease | Page 12 |
| Figure S7. Leave-one-out SNP analysis of lifetime smoking on lung cancer | Page 13 |
| Figure S8. Scatter plot of IVW and sensitivity analyses of lifetime smoking on schizophrenia | Page 14 |
| Figure S9. Scatter plot of IVW and sensitivity analyses of lifetime smoking on major depressive disorder | Page 15 |
| Figure S10. Scatter plot of IVW and sensitivity analyses of schizophrenia on lifetime smoking | Page 16 |
| Figure S11. Scatter plot of IVW and sensitivity analyses of depression on lifetime smoking | Page 17 |
| Figure S12. Single SNP analysis of lifetime smoking on schizophrenia | Page 18 |
| Figure S13. Single SNP analysis of lifetime smoking on depression | Page 19 |
| Figure S14. Single SNP analysis of schizophrenia on lifetime smoking | Page 20 |
| Figure S15. Single SNP analysis of depression on lifetime smoking | Page 21 |
| Figure S16. Leave-one-out analysis of lifetime smoking on schizophrenia | Page 22 |
| Figure S17. Leave-one-out analysis of lifetime smoking on depression | Page 23 |
| Figure S18. Leave-one-out analysis of schizophrenia on lifetime smoking | Page 24 |
| Figure S19. Leave-one-out analysis of depression on lifetime smoking | Page 25 |
|  |  |
| **Tables** |  |
| Table S1. SNPs associated with lifetime smoking index at the genome-wide level of significance | Page 26 |
| Table S2. Positive control two-sample MR to validate the lifetime smoking instrument  Table S3. Tests of the unweighted and weighted regression dilution I^2^_GX_ for the lifetime smoking exposure  Table S4. Tests of heterogeneity | Page 30  Page 31  Page 32 |
| Table S5. Test of directional pleiotropy using the MR Egger intercept  Table S6. Mendelian randomisation results following Steiger filtering. | Page 34  Page 35 |
| Table S7. Bi-directional two-sample Mendelian randomisation analyses of the effect of lifetime smoking on depression (2013).  Table S8. Sensitivity analysis without genotyping chip used as a covariate.  Table S9. Bi-directional two-sample Mendelian randomisation analyses of the effect of smoking on schizophrenia (2018).  Table S10. Bi-directional two-sample Mendelian randomisation analyses of the effect of smoking on schizophrenia (CHRNA5-A3-B4 Variants Removed)  Table S11. Bi-directional two-sample Mendelian randomisation analyses of lifetime smoking on depression (Howard et al., 2019 – ex UKBB and 23andMe)  Table S12. Comparison of Mendelian randomisation sensitivity methods | Page 36  Page 37  Page 38  Page 39  Page 40  Page 41 |
|  |  |
| **References** | Page 43 |

**Supplementary Note**

**Construction of the lifetime smoking index**

We simulated values of two constants: half-life ($\tau$), and lag time ($\delta$). Together these constants capture the non-linear risk of smoking on health. Half-life captures the exponentially decreasing effect of smoking at a given time on health outcomes. Lag time accounts for the observation that smokers are more at risk of certain diseases (e.g., lung cancer) immediately after stopping smoking than current smokers (Leffondré, Abrahamowicz, Siemiatycki, & Rachet, 2002). This is likely due to the prodromal consequences of disease being felt by the individual.

Simulations were run for possible values of τ (between 2 and 50 varying in increments of 1) and δ (between 0 and 5, varying in increments of 0.1) to find the best fitting model to explain the effects of lifetime smoking on lung cancer and all-cause mortality. Akaike information criterion (AIC) was used to select the best fitting model. The best fitting value for half-life was 18 for both lung cancer and all-cause mortality. We did not see any improvement of fit for changes in the value of lag time, therefore this was set to 0 as has been done previously (Lewer, McKee, Gasparrini, Reeves, & de Oliveira, 2017). This removes the effects of lag time from the model. These values were used to fit the final model which is:

tsc* = max(tsc - δ, 0)

dur* = max(dur + tsc - δ, 0) – tsc*

lifetime smoking = (1 – 0.5^dur*/τ^) (0.5^tsc*/τ^) ln(int+1)

…where τ = half-life, δ = lag time, int = cigarettes per day, tss = time started smoking, tsc = time since cessation, dur = duration of smoking (either age-tss for current smokers or [age-tsc]-tss for former smokers). So, in our case, where δ = 0, tsc* = tsc and consequently, our lifetime smoking calculation can be simplified to:

lifetime smoking = (1 – 0.5^dur/τ^) (0.5^tsc/τ^) ln(int+1)

Individuals who have never smoked and have no smoking exposure will have a value of 0. In our sample, values for smokers ranged from 0.007 (an individual who smoked 1 cigarette per day for 1 year) to 4.169 (an individual who currently smokes 140 a day and started smoking at age 11 years of age). Values of lifetime smoking were treated as continuous in subsequent analysis.

**Genotyping and exclusion procedure**

UK Biobank participants provided blood samples at initial assessment centre. Genotyping was performed using the Affymetrix UK BiLEVE Axiom array for 49,979 participants and using the Affymetrix UK Biobank Axiom® array for 438,398 participants. The two arrays share 95% coverage, but chip is adjusted for in all analyses because the UK BiLEVE sample is over represented for smokers. Imputation and initial quality control steps were performed by the Wellcome Trust Centre for Human Genetics resulting in over 90 million single nucleotide polymorphisms (SNPs) and indels (Bycroft et al., 2017).

Individuals were excluded if there were sex-mismatches between reported and chromosomal sex or aneuploidy (N=814). Individuals were restricted to European ancestry based on the first four principal components of population structure and related individuals were removed following MRC Integrative Epidemiology Unit filtering steps (Mitchell, Hemani, Dudding, & Paternoster, 2017). After excluding individuals who had withdrawn consent, 463,033 of the participants remained (Mitchell et al., 2017). We restricted our analysis to autosomes only and used filtering thresholds for SNPs of minor allele frequency (MAF) >0.01 and info score (measure of imputation uncertainty) >0.8.

**Instrument discovery for lifetime smoking index**

The results of our GWAS of lifetime smoking (N = 462,690) are presented in Figure 1 and Supplementary Figure S1. The most strongly associated regions on chromosome 15 (in the CHRNA5-A3-B4 gene complex) and chromosome 9 (near the DBH gene) have been previously shown to be associated with smoking heaviness and cessation respectively (Furberg et al., 2010). We identified 10,415 SNPs at the genome-wide level of significance (P < 5 × 10^-8^). After clumping and filtering, 126 independent SNPs remained. A full list of these SNPs and their z-scored effect sizes can be found in Supplementary Table S1.

**Instrument validation – positive control outcomes**

We tested our genetic instrument using the positive controls of lung cancer, CAD and DNA methylation at the AHRR locus. We conducted these analyses using GWAS summary data in a two-sample MR framework using our exposure instrument for lifetime smoking from our GWAS in the UK Biobank.

*Outcome GWAS samples for instrument validation*

For lung cancer, we used the summary data from the ILCCO consortium GWAS, which comprises 11,348 cases and 15,861 controls of European ancestry (Wang et al., 2014). Lung cancer cases were classified by tumour type using either the International Classification of Diseases for Oncology (ICD-O) or the World Health Organisation (WHO) coding. Tumours with overlapping histologies were classified as mixed. Most samples in the GWAS meta-analysis only included adenocarcinomas (AD) or squamous carcinomas (SQ) but classification was different for each contributing cohort. The GWAS was run with a binary outcome, case control status for any primary lung cancer tumour. For more details see Wang et al. (2014)^7^.

For CAD, we used the GWAS summary data from CARDIoGRAMplusC4D which comprises 60,801 cases and 123,504 controls of mixed ancestry (CARDIoGRAMplusC4D Consortium & others, 2015). Case status was defined as any CAD diagnosis including myocardial infarction, acute coronary syndrome, chronic stable angina or coronary stenosis of >50%. This GWAS was conducted with a binary outcome of CAD cases compared with controls.

We conducted a GWAS of AHRR (cg05575921) methylation in the Accessible Resource for Integrated Epigenomic Studies (ARIES) subset of ALSPAC (Relton et al., 2015). Genome-wide DNA methylation profiling in ARIES was performed using the Illumina Infinium HumanMethylation450 BeadChip (450K) array for ~1000 mother-offspring pairs (Relton et al., 2015). For this analysis, we used methylation data derived from whole blood which was collected from mothers (846 smokers and non-smokers) when the index offspring were around 18 years of age. Methylation data were normalised in R with the wateRmelon package (Pidsley et al., 2013) using the Touleimat and Tost (Touleimat & Tost, 2012)algorithm to reduce the non-biological differences between probes. As was done previously (Gaunt et al., 2016), AHRR methylation (cg05575921) was rank-normalised to remove outliers and regressed on the following covariates: age, the top ten ancestry principal components, bisulphite conversion batch and estimated white blood cell counts (using an algorithm based on differential methylation between cell types (Houseman et al., 2012)). Residuals were then taken forward and SNP association effects were obtained in PLINK1.07 using exact linear regression. Full details of the GWAS methods used for AHRR locus methylation are described elsewhere (Relton et al., 2015).

*Two-sample Mendelian randomisation of positive controls*

Analyses were conducted using MR Base, an R (R. Core Team, 2014) package for two-sample MR (Hemani et al., 2018). We compared results across five different MR methods: inverse-variance weighted, MR Egger (Bowden, Davey Smith, & Burgess, 2015), weighted median (Bowden, Davey Smith, Haycock, & Burgess, 2016), weighted mode (Hartwig, Smith, & Bowden, 2017) and MR RAPS (Zhao, Wang, Hemani, Bowden, & Small, 2018). Each method makes different assumptions and therefore a consistent effect across multiple methods strengthens causal evidence (Lawlor, Tilling, & Davey Smith, 2016). If a SNP was unavailable in the outcome GWAS summary statistics, then proxy SNPs were searched for with a minimum LD r^2^ = 0.8. We aligned palindromic SNPs with MAF<0.3.

*Results*

We validated the genetic instrument for lifetime smoking exposure using two-sample MR of smoking on positive control outcomes: lung cancer, CAD and hypomethylation at the *AHRR* locus. All five MR methods indicated the expected direction of effect (see Supplementary Table S2) increasing risk of disease outcomes and decreasing *AHRR* methylation, with the exception of the MR Egger SIMEX adjusted estimates for CAD. However, these should be interpreted with caution given the low I^2^_GX_ (see Supplementary Table S3) (Bowden, Del Greco M, et al., 2016). There was strong evidence of an effect of lifetime smoking on increased odds of lung cancer and CAD. There was weaker evidence of an effect on AHRR methylation, possibly due to lower power. Sensitivity analyses are presented in Supplementary Figures S2-S7. There was evidence of significant heterogeneity in the SNP-exposure effects (see Supplementary Table S3); however, tests of MR Egger intercepts generally indicated weak evidence of directional pleiotropy (see Supplementary Table S4), with the exception of CAD.

**Instrument validation – replication in an independent sample**

*ALSPAC sample description and measures*

To test prediction in an independent sample, we used 2,712 mothers from Avon Longitudinal Study of Parents and Children (ALSPAC). ALSPAC is a longitudinal birth cohort, which recruited 14,541 pregnant women between April 1991 and December 1992 with detailed descriptions reported elsewhere (Boyd et al., 2013; Fraser et al., 2012). The study website contains details of all the data that is available through a fully searchable data dictionary and variable search tool ([www.bristol.ac.uk/alspac/researchers/our-data/](http://www.bristol.ac.uk/alspac/researchers/our-data/)). Self-reported measures of smoking status, age at initiation, age at cessation and cigarettes per day were collected from these women when their offspring were 18 years of age (mean mothers age = 48 years, SD = 4.3).

*Statistical Analysis*

PLINK was used to generate a polygenic risk score in ALSPAC (Purcell et al., 2007). Linear regression was used to estimate the percentage variance of lifetime smoking explained by the polygenic score.

*Results*

In the ALSPAC independent sample, the 126 SNPs explained 0.36% of the variance in lifetime smoking (P = 0.002).

**Genetic Correlations**

*Methods*

Genetic correlations were first calculated between lifetime smoking, smoking initiation (Liu et al., 2019), depression (Wray, Sullivan, & others, 2018) and schizophrenia (Ripke et al., 2014) using the summary statistics outlined in the main text. Full summary statistics were only available for depression and smoking initiation excluding 23andMe. Genetic correlations were calculated using the LD Score Regression software (Bulik-Sullivan et al., 2015).

*Results*

There was evidence of positive genetic correlations between both smoking phenotypes (rG = 0.868, <0.001), between lifetime smoking and depression (rG = 0.404, p<0.001) and between lifetime smoking and schizophrenia (rG = 0.160, p<0.001). Genetic correlations between smoking initiation and mental health are reported elsewhere (Liu et al., 2019). There was a positive genetic correlation between smoking initiation and schizophrenia (rG = 0.137, p<0.001) and between smoking initiation and major depression (rG = 0.186, p<0.001).

**Figure 1. Quantile-quantile plot of SNP associations with lifetime smoking index.**

*
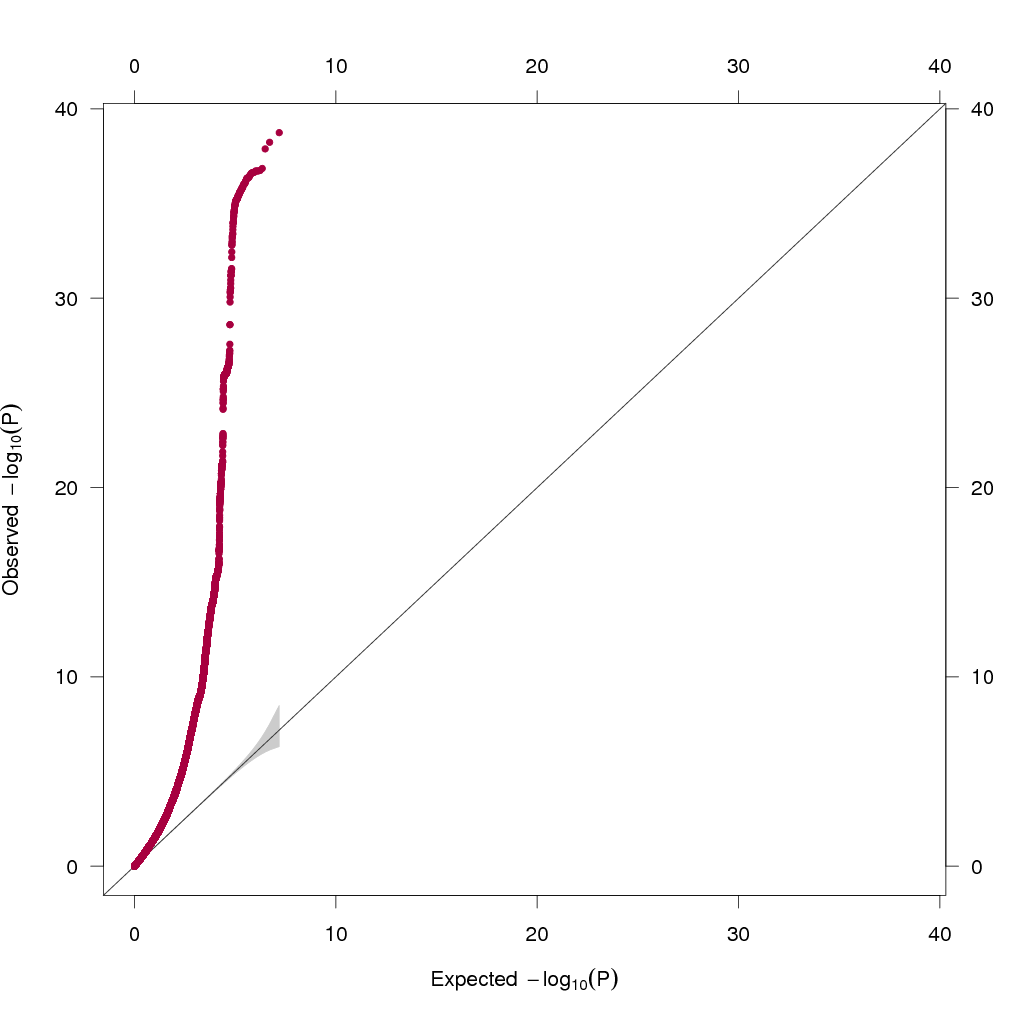
*

The x-axis indicates the -log_10_ p-value expected under the null hypothesis and the y-axis represents the observed -log_10_ p-value.

Testing inflation by population structure

The QQ plot suggests evidence of inflation due to population stratification. To check this, we calculated the LD score intercept (1.051, SE = 0.0094) and mean chi-square statistic (1.907) which give an attenuation ratio of 0.056, minimal evidence of inflation. The pattern seen here is therefore likely due to the large sample size giving power to detect high numbers of associations.

**Figure S2. Scatter plot of IVW and sensitivity analyses of lifetime smoking on coronary artery disease.**

*
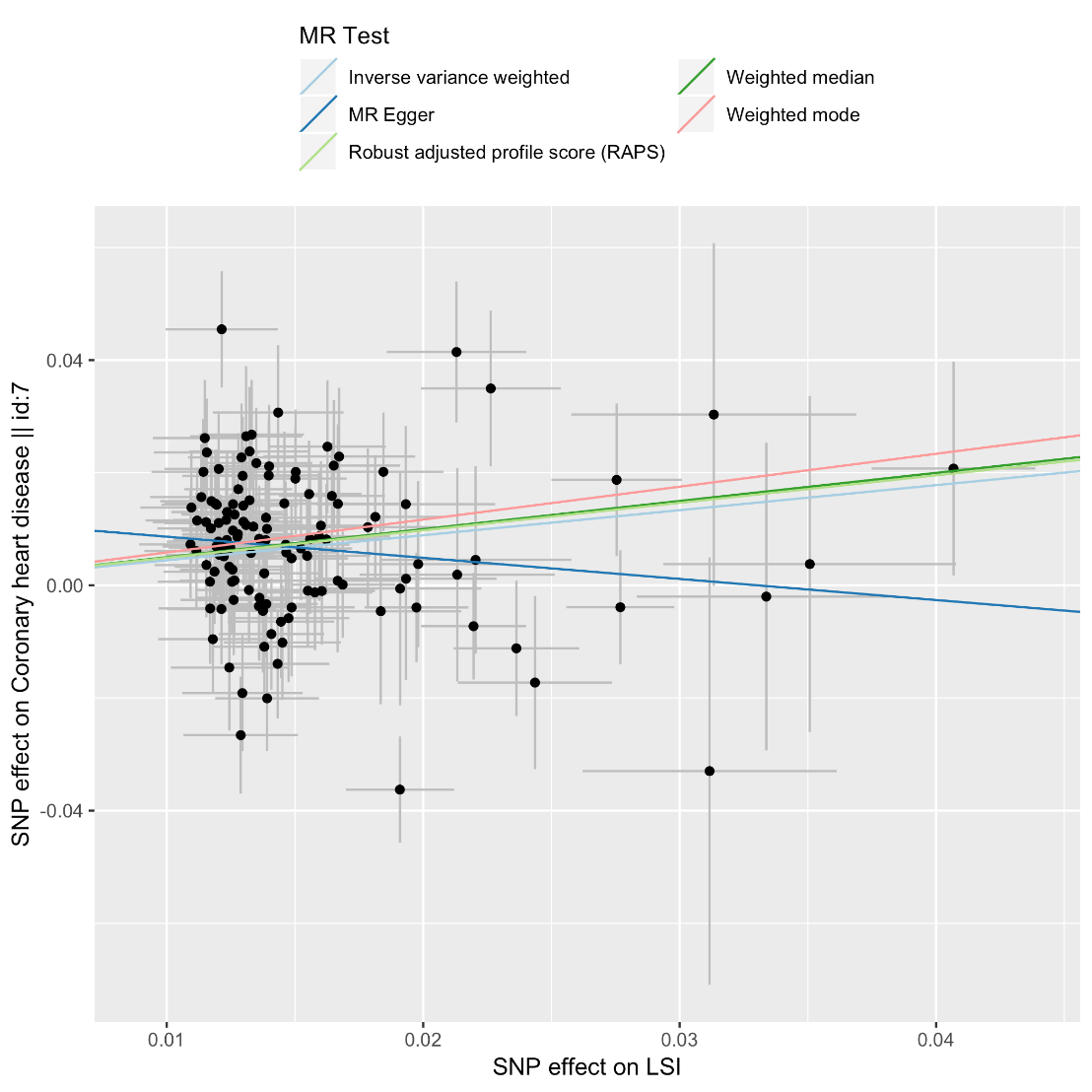
*

**Figure S3. Scatter plot of IVW and sensitivity analyses of lifetime smoking on lung cancer.**

*
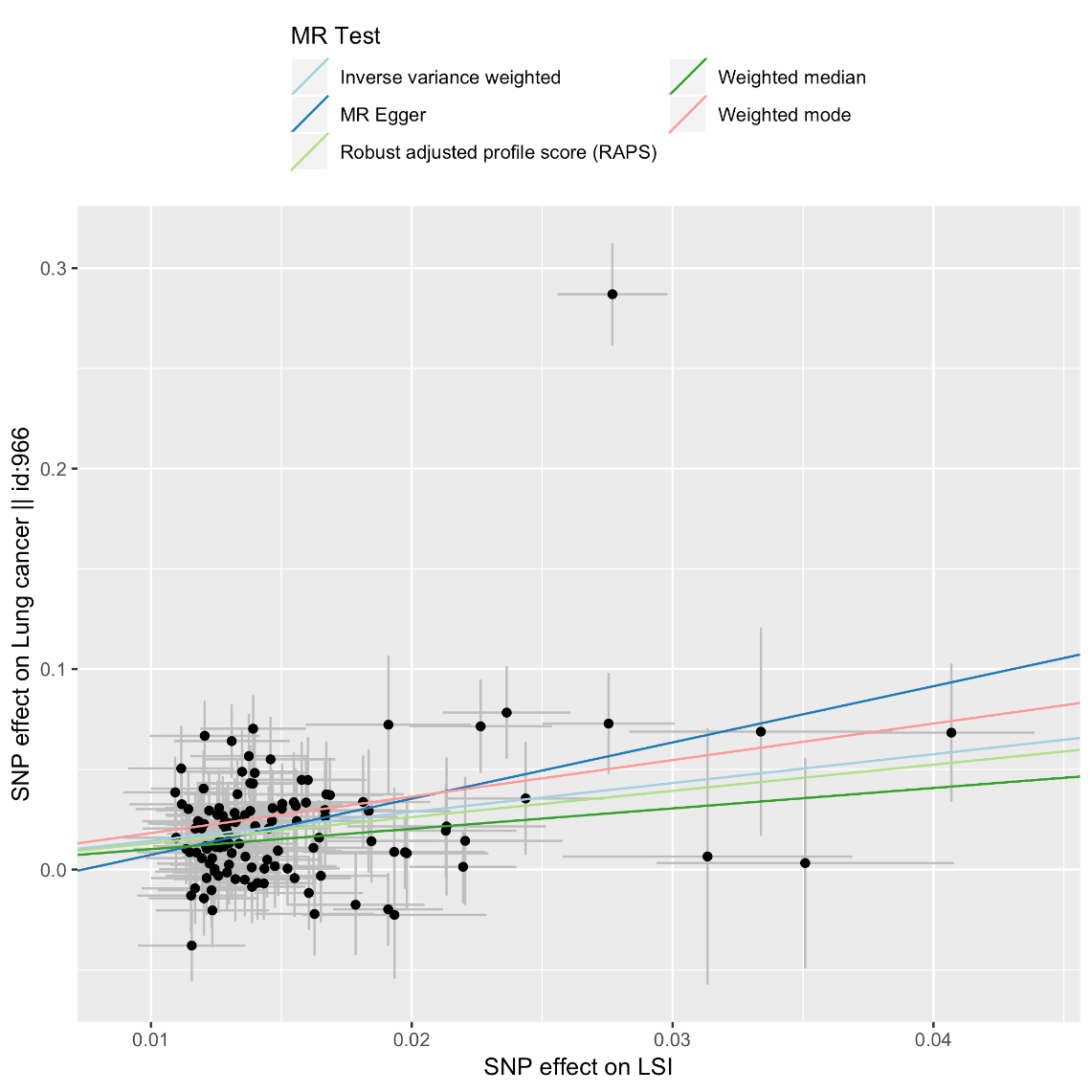
*

Figure S3 shows evidence of an outlier, however this plot does not account for SE in the SNP effect. Therefore, we followed up this outlier using radial MR.

Radial MR

A radial MR analysis of lifetime smoking on lung cancer using first order weights and an alpha level of 0.01 identified 5 outliers for the IVW method (in order of how large an outlier they are):

| **IVW Outliers** |
| --- |
| 1. rs10918701 |
| 2. rs12244388 |
| 3. rs329120  4. rs6562474 |
| 5. rs8042849 |
|  |
|  |

Outliers are usually removed in an incremental fashion, beginning with the largest. However, we can see from our leave-one-out analysis (see Figure S7) that the two largest outliers for both methods (rs10918701 and rs12244388) do not affect the estimate once removed.

**Figure S4. Single SNP effects of lifetime smoking on coronary artery disease.**


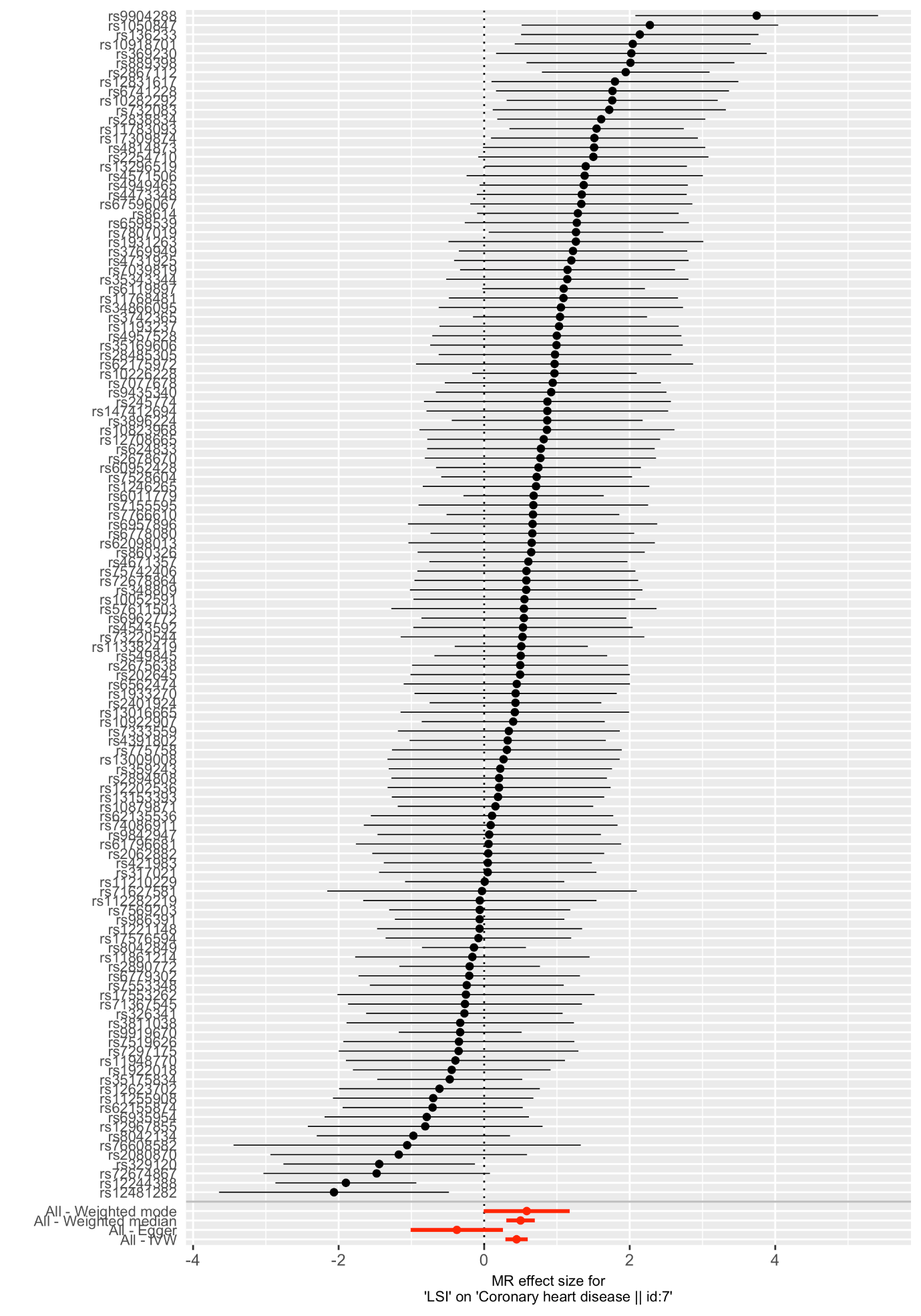


**Figure S5. Single SNP effects of lifetime smoking on lung cancer.**


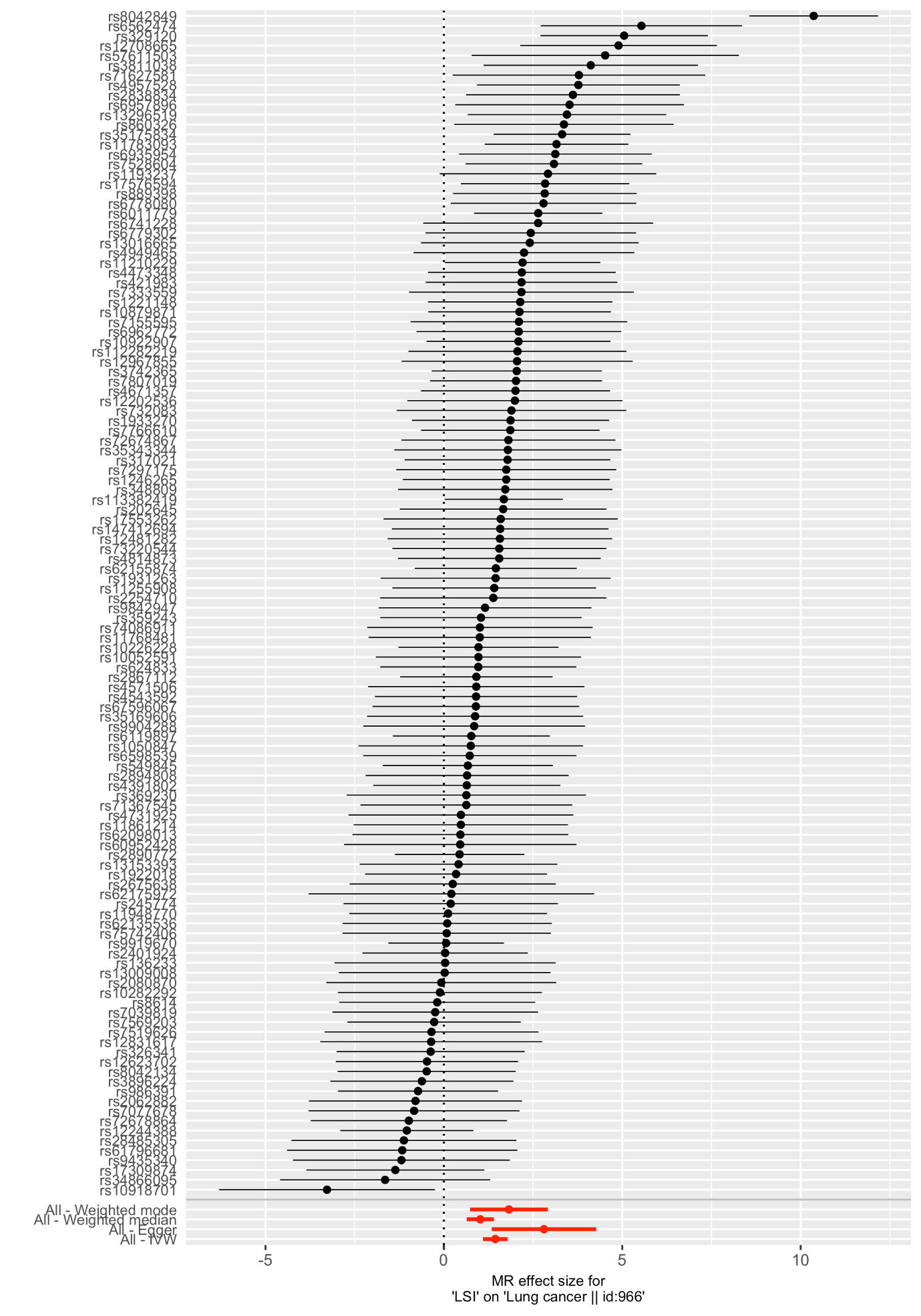


The SNP with the largest effect is rs8042849, which is an intron variant of the *HYKK* gene, has previously been associated with nicotine dependence and forced expiratory volume (FEV).

**Figure S6. Leave-one-out effects of lifetime smoking on coronary artery disease using the inverse-variance weighted method.**

*
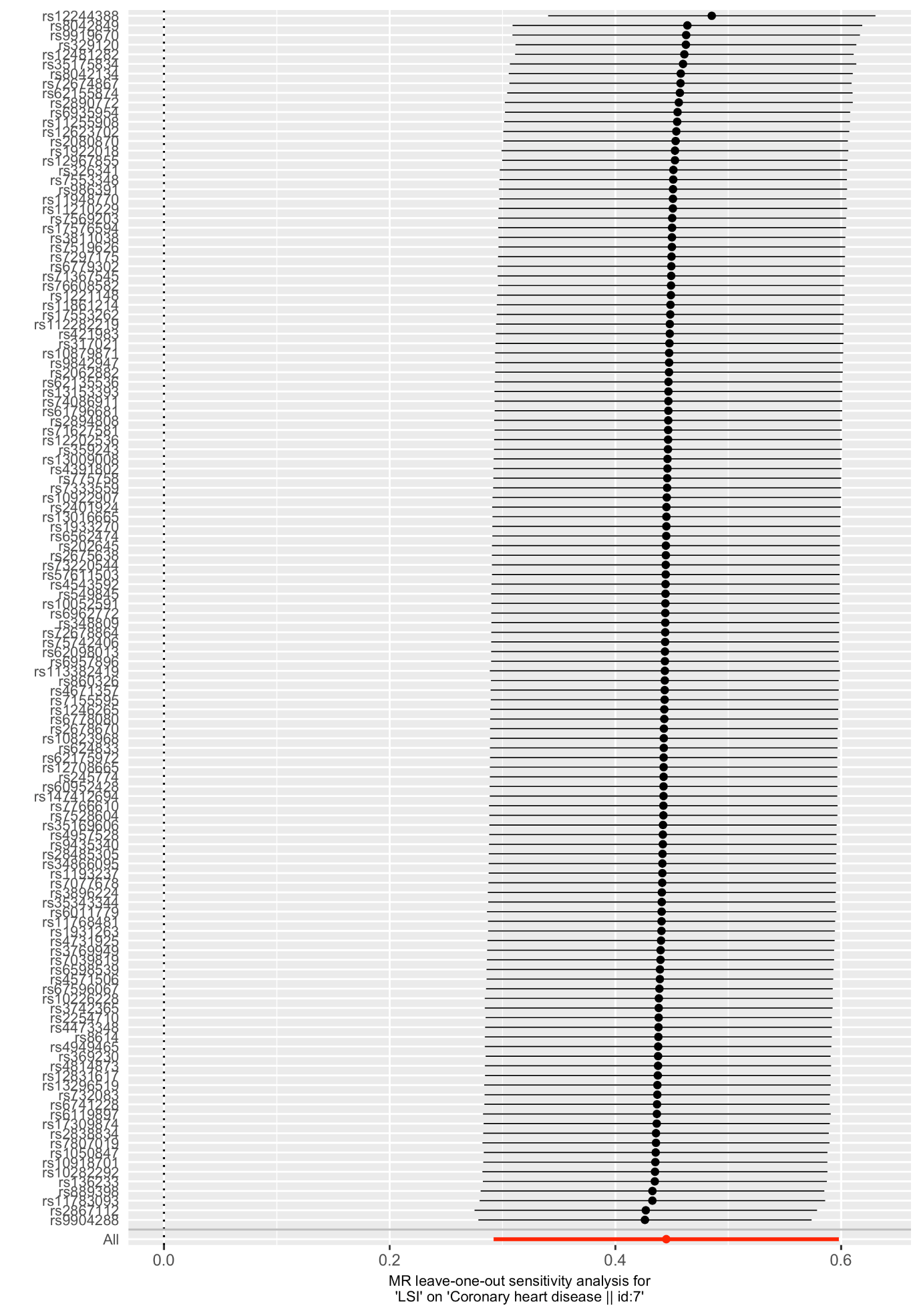
*

**Figure S7. Leave-one-out effects of lifetime smoking on lung cancer using the inverse-variance weighted method.**


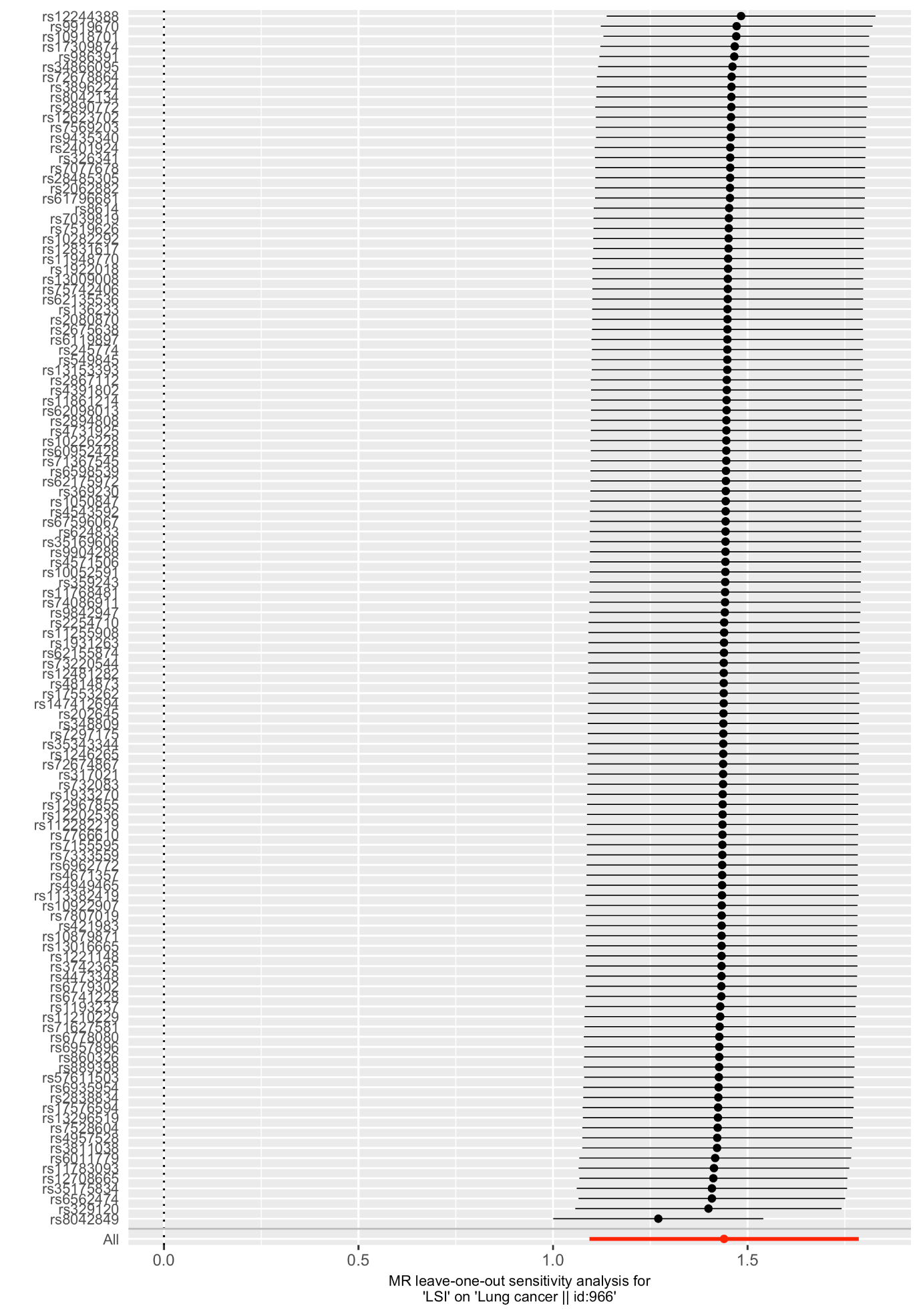


**Figure S8. Scatter plot of IVW and sensitivity analyses of lifetime smoking on schizophrenia.**

*
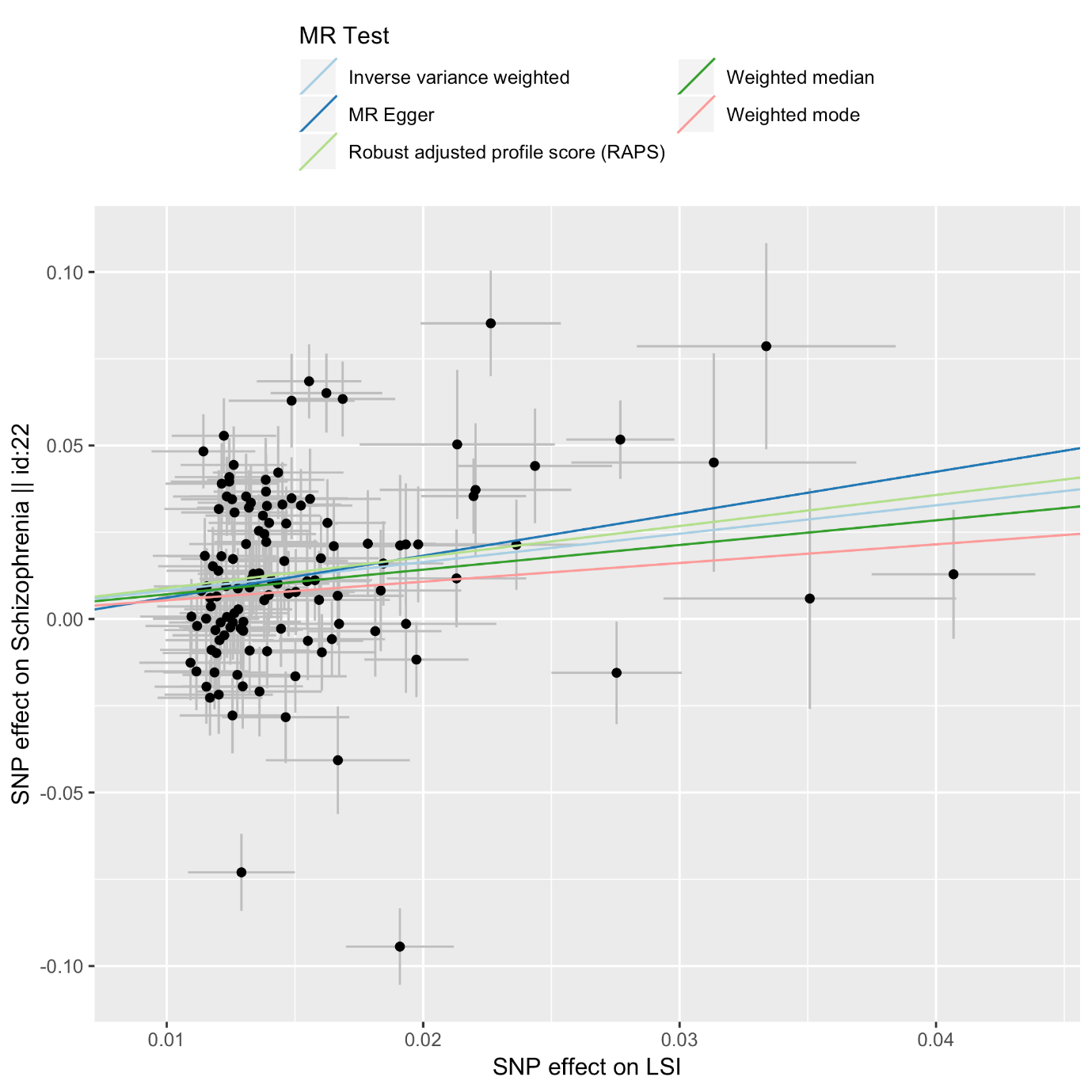
*

**Figure S9. Scatter plot of IVW and sensitivity analyses of lifetime smoking on major depressive disorder.**

*
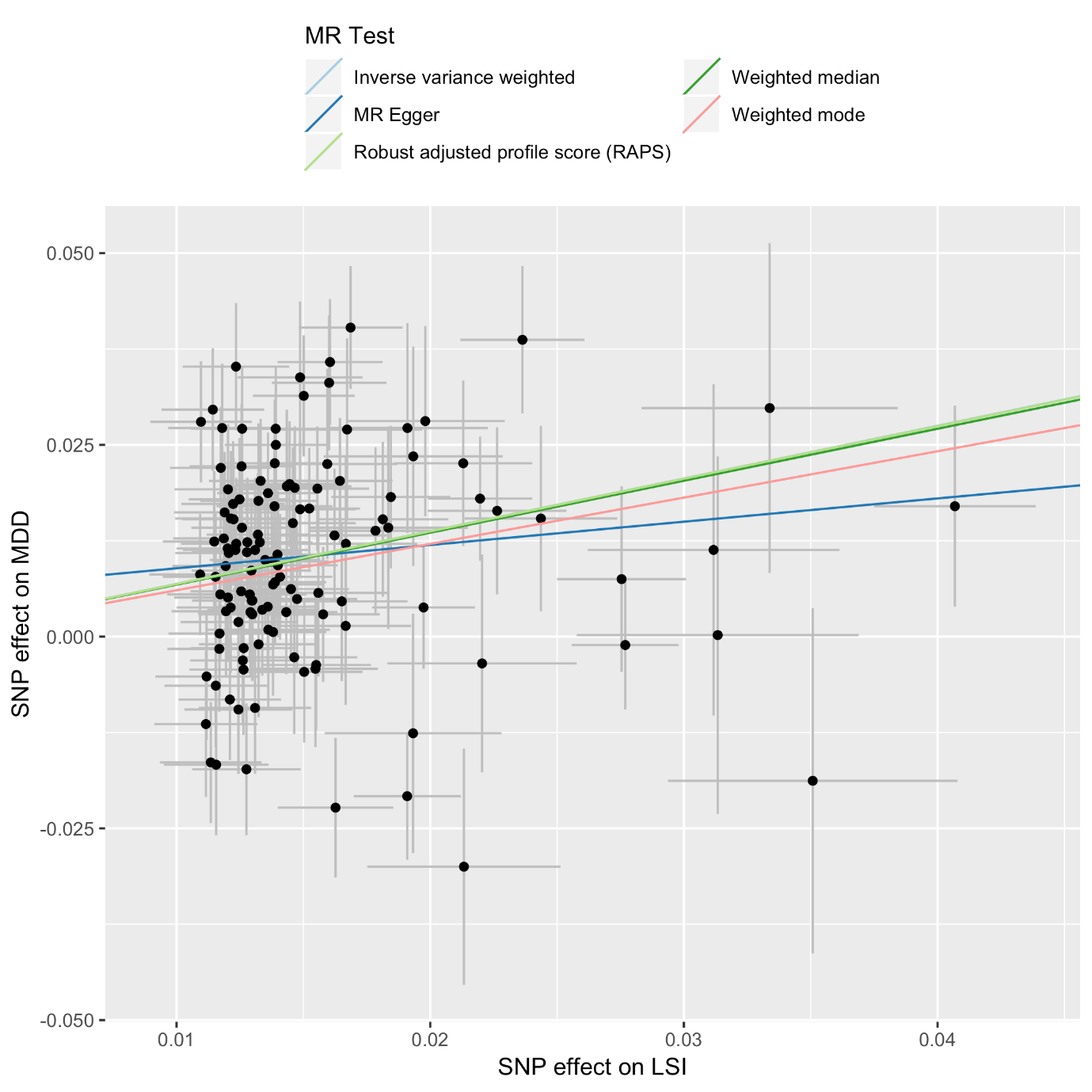
*

**Figure S10. Scatter plot of IVW and sensitivity analyses of schizophrenia on lifetime smoking.**

*
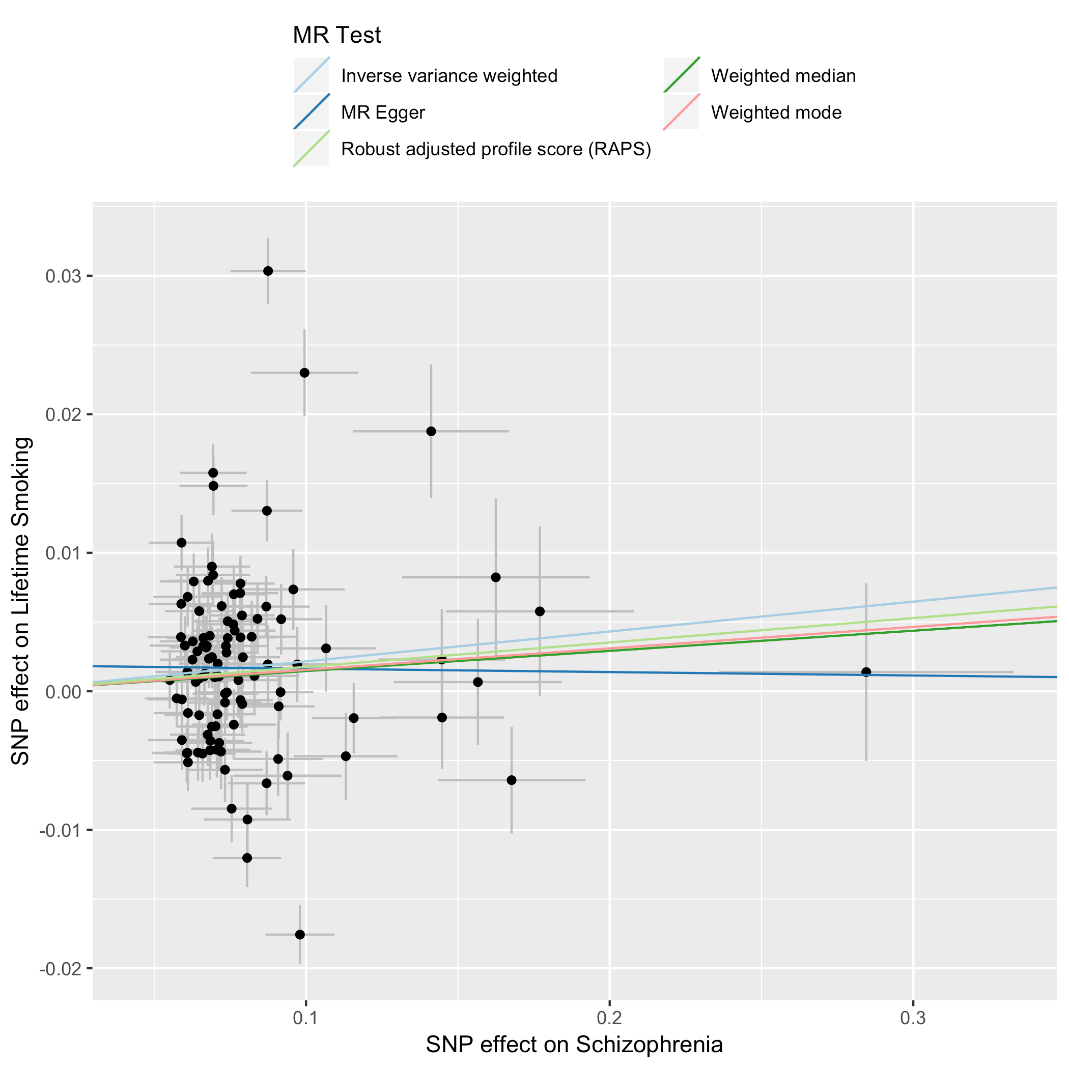
*

**Figure S11. Scatter plot of IVW and sensitivity analyses of depression on lifetime smoking.**

**
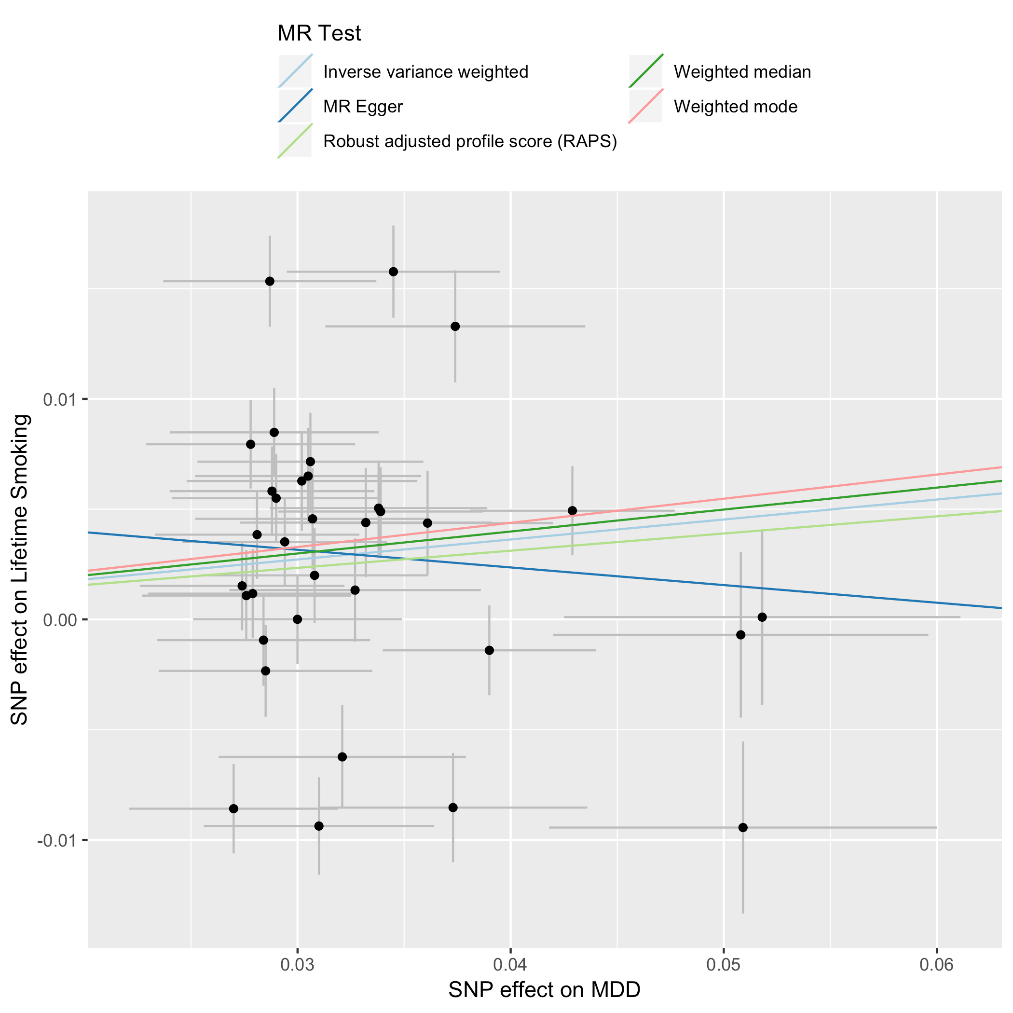
**

**Figure S12. Single SNP analysis of lifetime smoking on schizophrenia.**

*
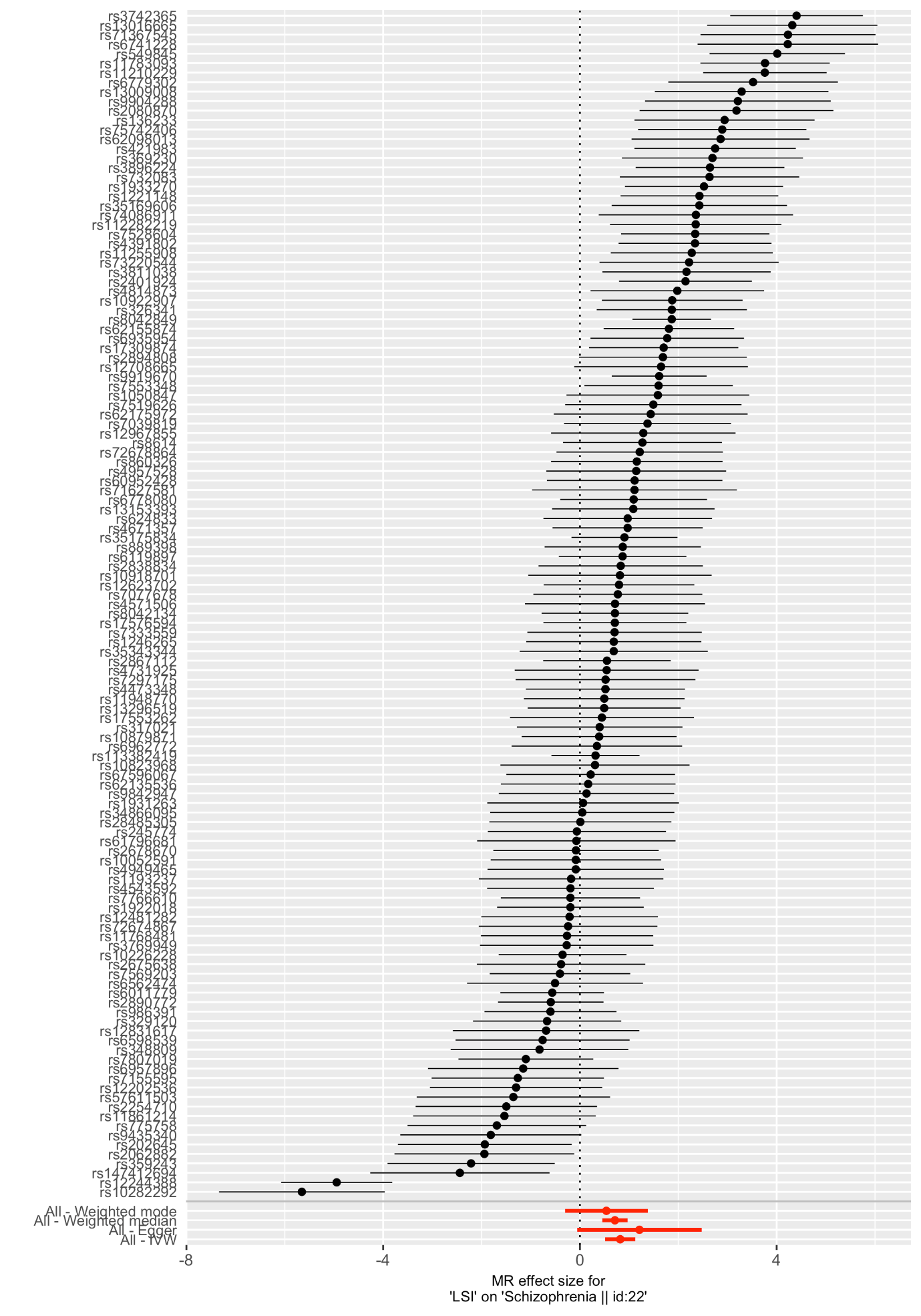
*

**Figure S13. Single SNP analysis of lifetime smoking on depression.**

*
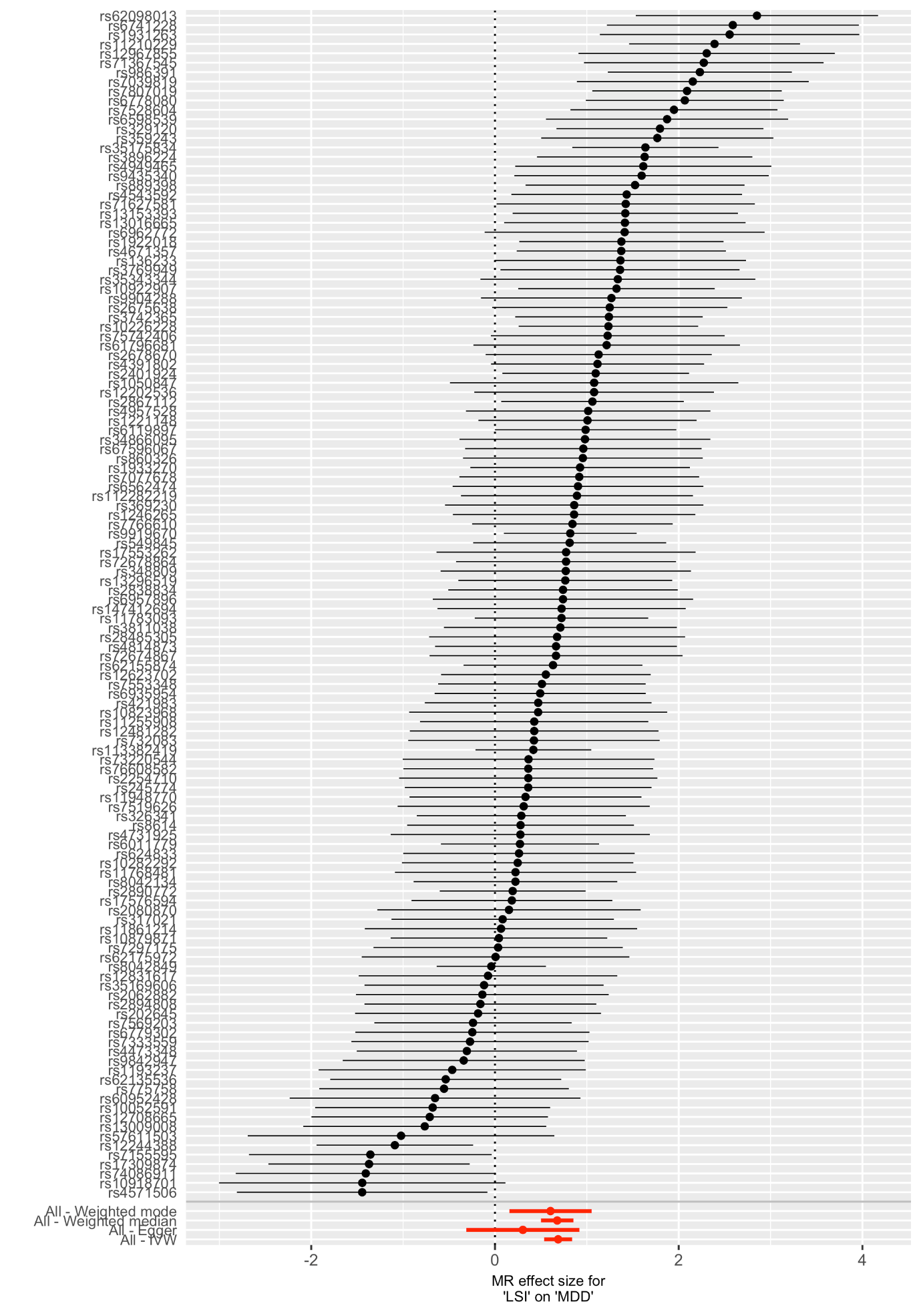
*

**Figure S14. Single SNP analysis of schizophrenia on lifetime smoking.**

**
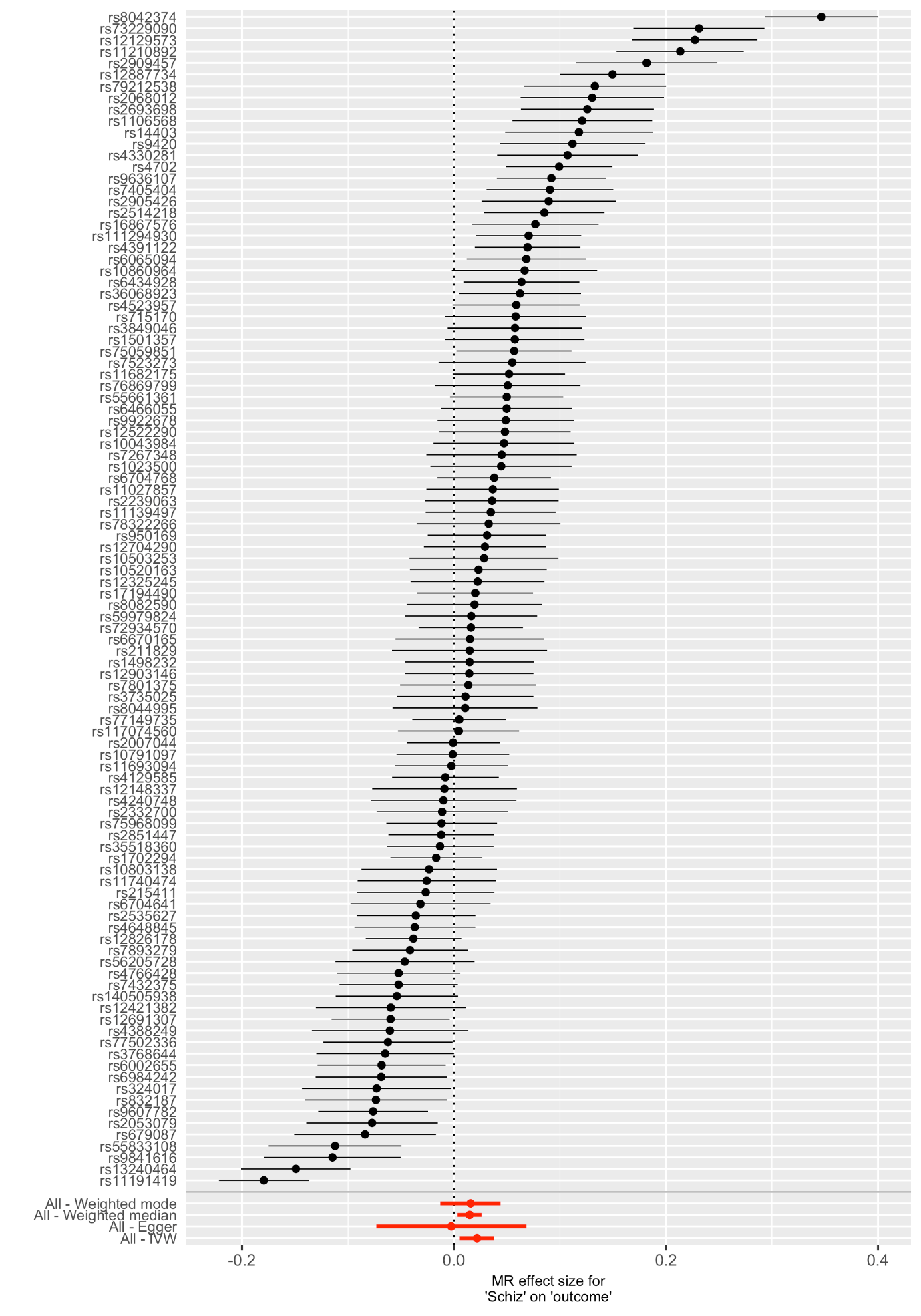
**

The SNP with the largest effect on smoking is from rs8042374 located in the CHRNA3 gene (known to be associated with Nicotine dependence (Munafò et al., 2012; Thorgeirsson et al., 2008; Tobacco Consortium, 2010; Ware, van den Bree, & Munafò, 2011). However, removing this SNP did not remove the effect (see Supplementary Figure S18).

**Figure S15. Single SNP analysis of depression on lifetime smoking.**

*
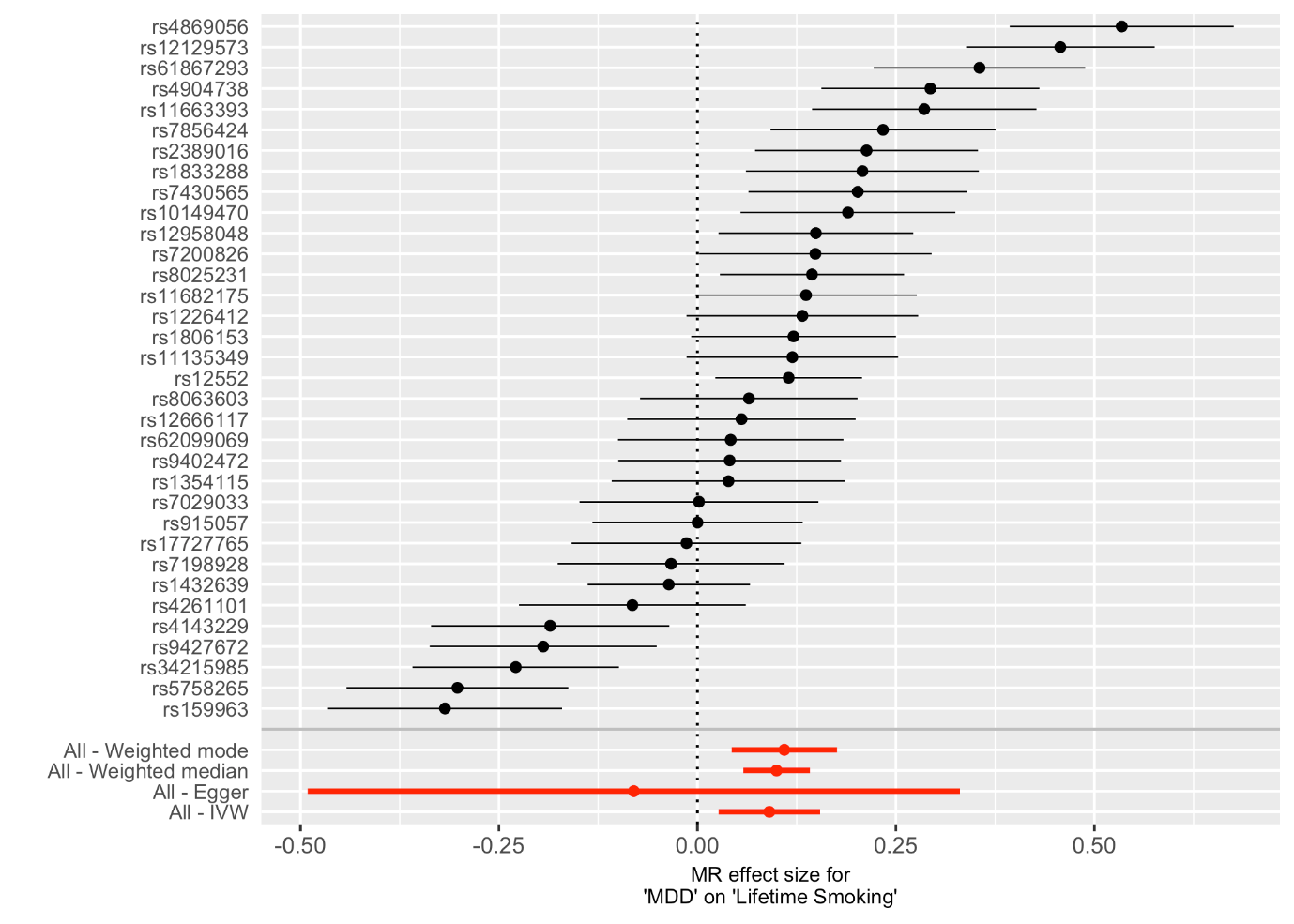
*

**Figure S16. Leave-one-out analysis of lifetime smoking on schizophrenia.**

*
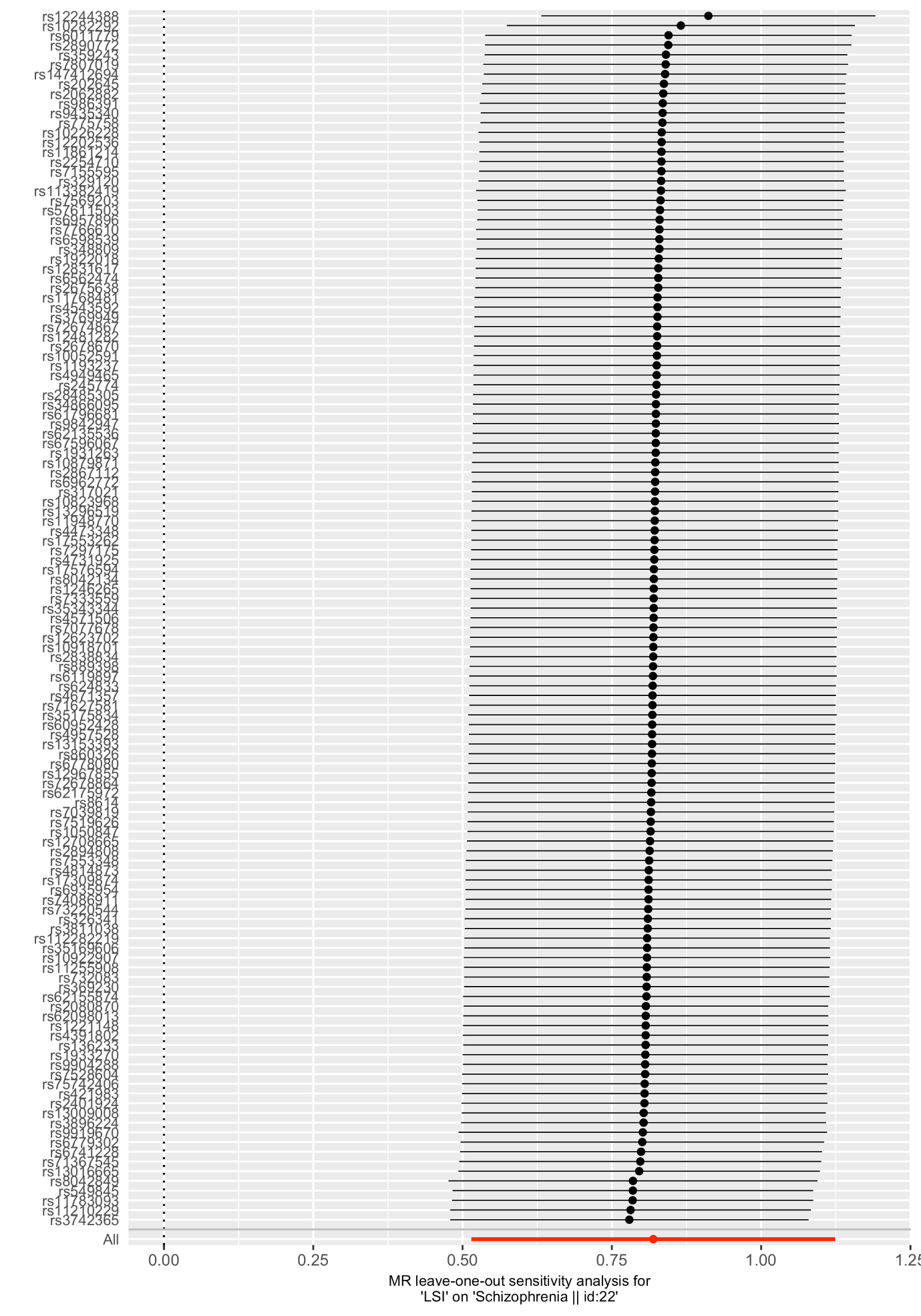
*

**Figure S17. Leave-one-out analysis of lifetime smoking on depression.**

*
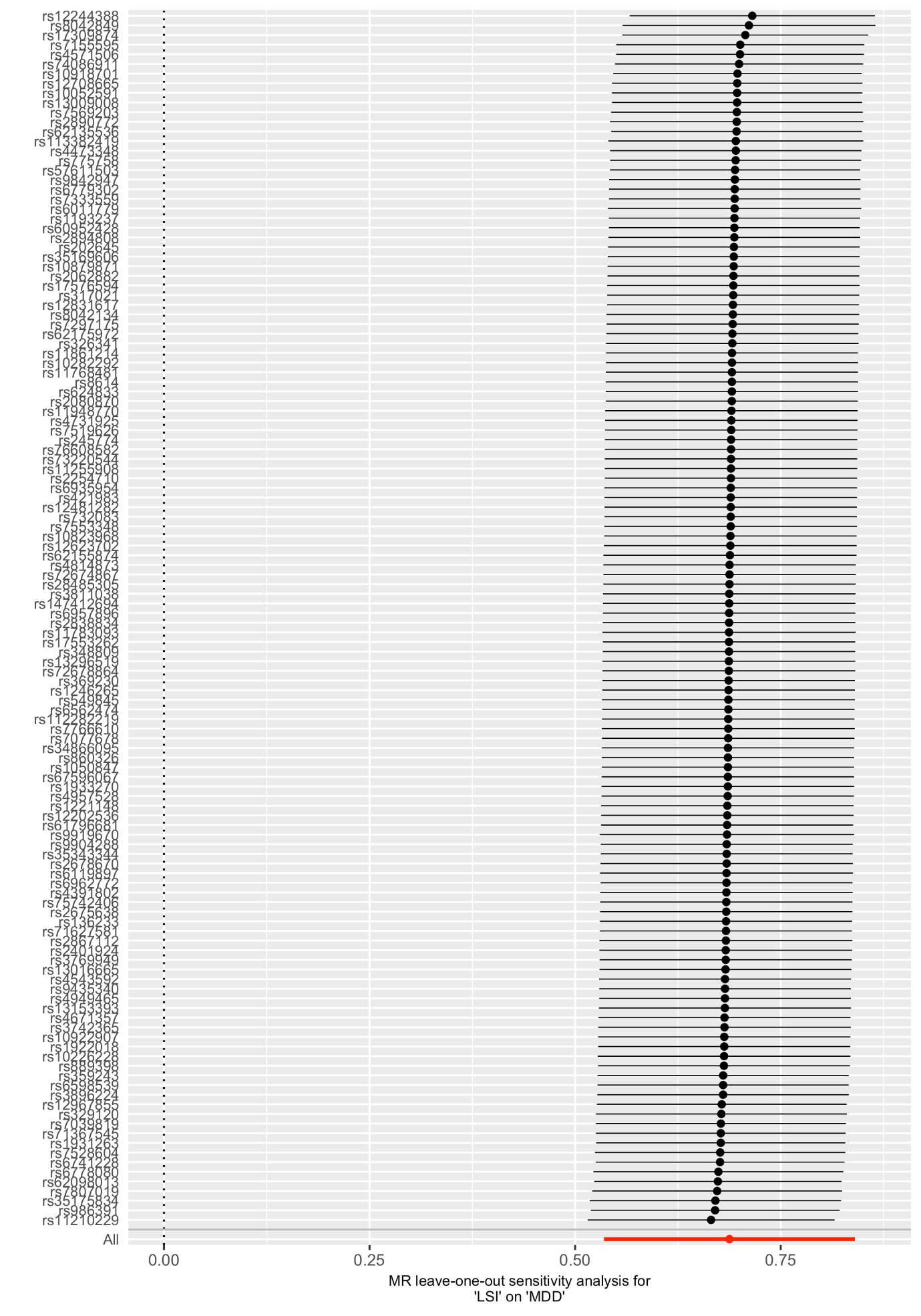
*

**Figure 18. Leave-one-out analysis of schizophrenia on lifetime smoking.**

**
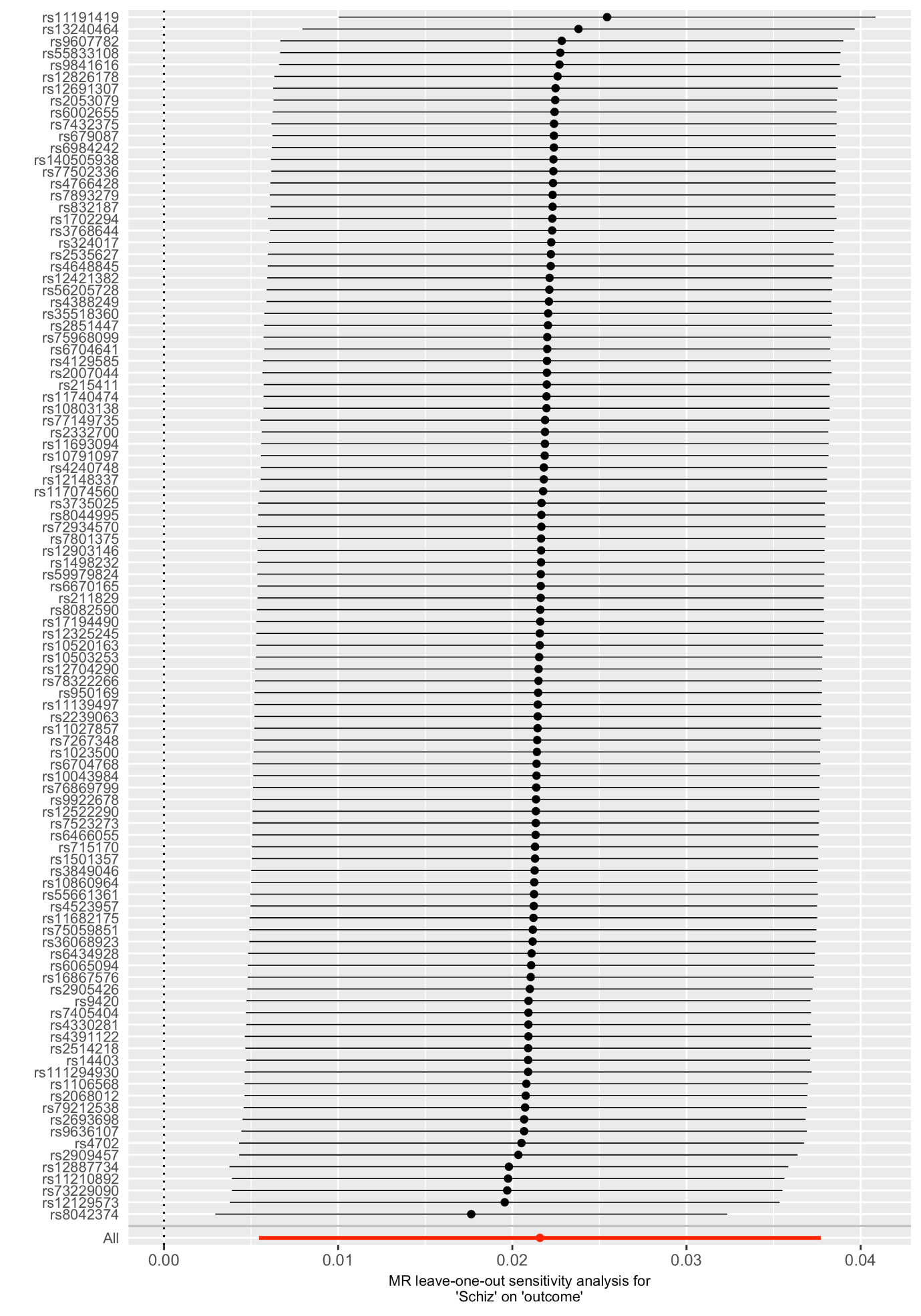
**

**Figure S19. Leave-one-out analysis of depression on lifetime smoking.**


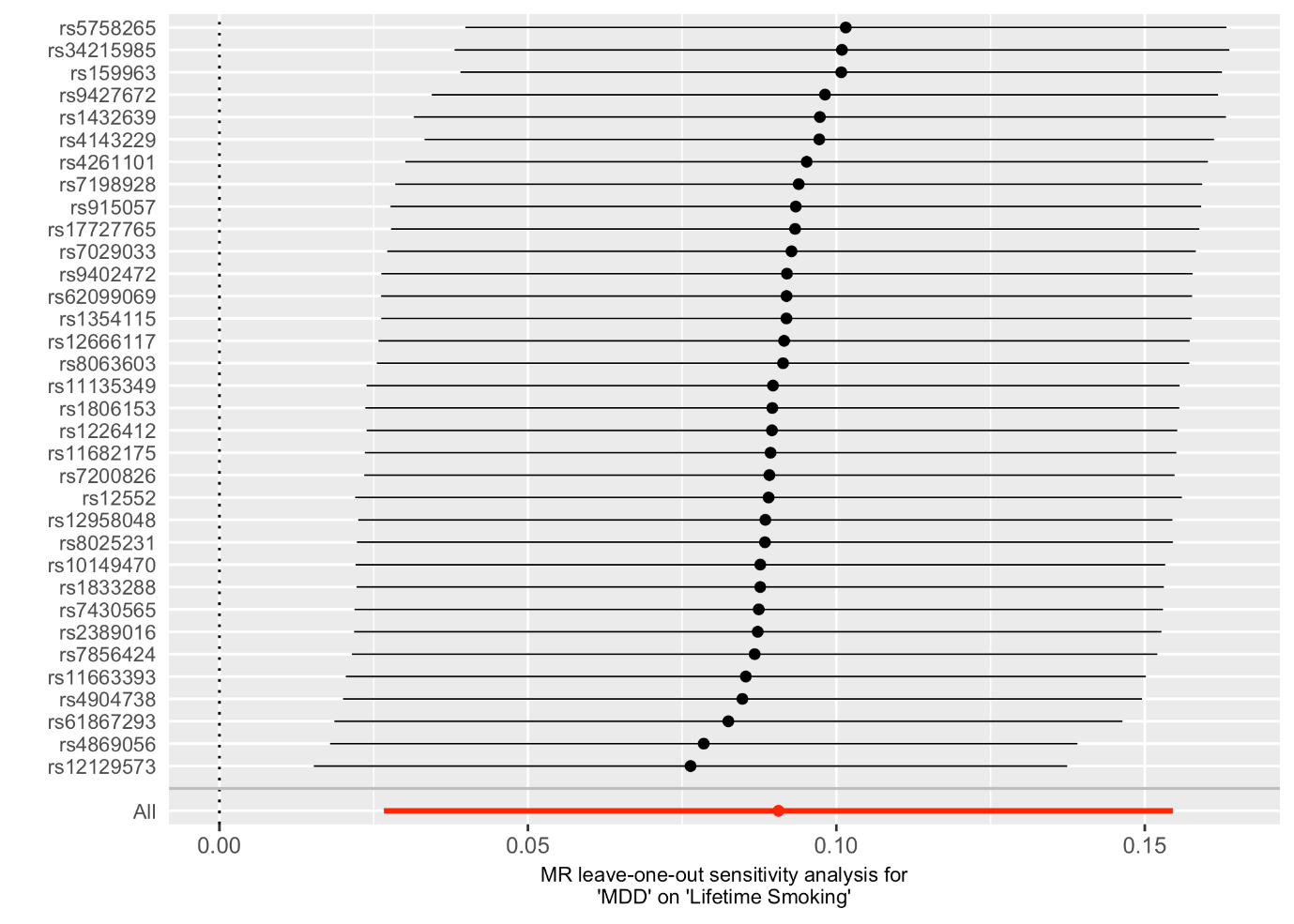


**Table S1. SNPs associated with lifetime smoking index at the genome-wide level of significance (p<5x10^-8^) and clumped for independence at kb=10000 and r^2^=0.001, in ascending order of p-value.**

| **SNP** | **CHR** | **BP** | **EA** | **NonEA** | **EAF** | **Beta** | **SE** | **p-value** |
| --- | --- | --- | --- | --- | --- | --- | --- | --- |
| rs8042849 | 15 | 78817929 | C | T | 0.342 | 0.028 | 0.002 | 1.80E-39 |
| rs113382419 | 9 | 1.36E+08 | C | A | 0.889 | -0.041 | 0.003 | 3.00E-37 |
| rs6011779 | 20 | 61984317 | C | T | 0.191 | 0.028 | 0.003 | 2.30E-27 |
| rs9919670 | 11 | 1.13E+08 | G | A | 0.612 | -0.022 | 0.002 | 7.60E-27 |
| rs2890772 | 2 | 1.46E+08 | G | T | 0.413 | -0.020 | 0.002 | 2.10E-22 |
| rs35175834 | 15 | 47680815 | G | A | 0.788 | -0.024 | 0.002 | 4.60E-22 |
| rs12244388 | 10 | 1.05E+08 | G | A | 0.661 | -0.019 | 0.002 | 1.40E-19 |
| rs11783093 | 8 | 27425349 | C | T | 0.839 | 0.023 | 0.003 | 1.20E-16 |
| rs11210229 | 1 | 73860028 | A | G | 0.384 | 0.017 | 0.002 | 2.00E-16 |
| rs62155874 | 2 | 1.06E+08 | A | G | 0.873 | -0.024 | 0.003 | 5.20E-16 |
| rs10226228 | 7 | 32315613 | A | G | 0.630 | -0.016 | 0.002 | 2.00E-15 |
| rs6119897 | 20 | 31145415 | G | A | 0.762 | -0.018 | 0.002 | 3.60E-15 |
| rs2867112 | 2 | 651349 | T | G | 0.835 | 0.021 | 0.003 | 4.80E-15 |
| rs986391 | 5 | 1.67E+08 | G | A | 0.367 | 0.016 | 0.002 | 9.40E-15 |
| rs3742365 | 14 | 1.04E+08 | T | C | 0.595 | -0.016 | 0.002 | 2.50E-14 |
| rs2401924 | 7 | 1.15E+08 | G | C | 0.502 | 0.015 | 0.002 | 2.70E-14 |
| rs7807019 | 7 | 1.18E+08 | A | G | 0.540 | -0.015 | 0.002 | 6.70E-14 |
| rs549845 | 1 | 44076469 | G | A | 0.301 | 0.016 | 0.002 | 8.30E-14 |
| rs10922907 | 1 | 91193049 | A | T | 0.451 | 0.015 | 0.002 | 3.00E-13 |
| rs7569203 | 2 | 45154418 | A | C | 0.689 | -0.016 | 0.002 | 7.40E-13 |
| rs17309874 | 11 | 27667236 | G | A | 0.740 | -0.016 | 0.002 | 9.70E-13 |
| rs6778080 | 3 | 49317338 | T | C | 0.267 | 0.016 | 0.002 | 1.30E-12 |
| rs8042134 | 15 | 97514404 | T | G | 0.541 | -0.014 | 0.002 | 1.30E-12 |
| rs17576594 | 4 | 1.48E+08 | G | A | 0.724 | 0.016 | 0.002 | 1.70E-12 |
| rs7766610 | 6 | 1.12E+08 | C | A | 0.183 | 0.018 | 0.003 | 2.20E-12 |
| rs1922018 | 7 | 3560401 | C | T | 0.364 | 0.014 | 0.002 | 3.00E-12 |
| rs7553348 | 1 | 75005067 | G | A | 0.438 | 0.014 | 0.002 | 5.20E-12 |
| rs7528604 | 1 | 66407352 | G | A | 0.566 | 0.014 | 0.002 | 5.70E-12 |
| rs329120 | 5 | 1.34E+08 | C | T | 0.581 | 0.014 | 0.002 | 6.30E-12 |
| rs12623702 | 2 | 2.03E+08 | A | G | 0.613 | -0.014 | 0.002 | 7.70E-12 |
| rs13296519 | 9 | 1.28E+08 | G | T | 0.606 | -0.014 | 0.002 | 8.10E-12 |
| rs6935954 | 6 | 26255451 | A | G | 0.421 | 0.014 | 0.002 | 8.20E-12 |
| rs4671357 | 2 | 60136176 | T | C | 0.519 | -0.014 | 0.002 | 1.10E-11 |
| rs3896224 | 10 | 1.06E+08 | A | G | 0.585 | 0.014 | 0.002 | 1.10E-11 |
| rs326341 | 3 | 1.08E+08 | G | A | 0.525 | 0.014 | 0.002 | 1.20E-11 |
| rs4391802 | 11 | 28674592 | A | G | 0.707 | 0.015 | 0.002 | 1.40E-11 |
| rs72678864 | 4 | 1.12E+08 | G | A | 0.829 | 0.018 | 0.003 | 1.60E-11 |
| rs112282219 | 11 | 46632809 | G | A | 0.959 | -0.033 | 0.005 | 3.80E-11 |
| rs10879871 | 12 | 75380511 | T | G | 0.343 | -0.014 | 0.002 | 5.00E-11 |
| rs889398 | 16 | 69556715 | C | T | 0.588 | 0.013 | 0.002 | 6.30E-11 |
| rs4473348 | 2 | 1.82E+08 | A | T | 0.250 | -0.015 | 0.002 | 6.40E-11 |
| rs1221148 | 9 | 1.22E+08 | C | G | 0.587 | 0.013 | 0.002 | 7.30E-11 |
| rs317021 | 4 | 35418368 | T | A | 0.814 | -0.017 | 0.003 | 1.10E-10 |
| rs1933270 | 1 | 49977965 | T | G | 0.364 | 0.013 | 0.002 | 1.50E-10 |
| rs8614 | 17 | 27588806 | C | A | 0.817 | -0.017 | 0.003 | 1.80E-10 |
| rs11255908 | 10 | 8802912 | T | G | 0.743 | -0.015 | 0.002 | 2.30E-10 |
| rs13153393 | 5 | 1.68E+08 | A | G | 0.884 | -0.020 | 0.003 | 2.50E-10 |
| rs2678670 | 2 | 1.04E+08 | A | T | 0.486 | 0.013 | 0.002 | 3.10E-10 |
| rs7333559 | 13 | 1.01E+08 | G | A | 0.212 | 0.015 | 0.002 | 3.20E-10 |
| rs76608582 | 19 | 4474725 | C | A | 0.953 | 0.031 | 0.005 | 3.20E-10 |
| rs421983 | 3 | 84892866 | T | C | 0.519 | 0.013 | 0.002 | 3.30E-10 |
| rs4543592 | 9 | 3014254 | T | C | 0.520 | -0.012 | 0.002 | 4.50E-10 |
| rs11948770 | 5 | 13246336 | T | C | 0.768 | -0.015 | 0.002 | 4.90E-10 |
| rs7039819 | 9 | 82430418 | G | A | 0.427 | 0.013 | 0.002 | 5.10E-10 |
| rs10282292 | 7 | 1.11E+08 | C | T | 0.362 | 0.013 | 0.002 | 5.90E-10 |
| rs2838834 | 21 | 46665208 | C | T | 0.699 | -0.013 | 0.002 | 6.30E-10 |
| rs624833 | 4 | 2881256 | T | G | 0.695 | 0.013 | 0.002 | 6.60E-10 |
| rs62135536 | 2 | 44326028 | C | T | 0.968 | 0.035 | 0.006 | 8.00E-10 |
| rs3811038 | 2 | 1.13E+08 | T | C | 0.724 | -0.014 | 0.002 | 8.90E-10 |
| rs359243 | 2 | 60475509 | T | C | 0.393 | -0.013 | 0.002 | 9.50E-10 |
| rs11768481 | 7 | 96629103 | C | A | 0.666 | 0.013 | 0.002 | 9.90E-10 |
| rs6779302 | 3 | 16859710 | G | T | 0.633 | -0.013 | 0.002 | 1.20E-09 |
| rs35169606 | 8 | 9604066 | T | G | 0.612 | 0.013 | 0.002 | 1.20E-09 |
| rs67596067 | 17 | 50333733 | G | A | 0.649 | -0.013 | 0.002 | 1.20E-09 |
| rs2675638 | 10 | 63576286 | G | A | 0.581 | 0.012 | 0.002 | 1.30E-09 |
| rs75742406 | 11 | 17070365 | G | A | 0.739 | 0.014 | 0.002 | 1.30E-09 |
| rs71367545 | 18 | 77576337 | G | A | 0.791 | -0.015 | 0.002 | 1.40E-09 |
| rs71627581 | 5 | 43161351 | G | A | 0.889 | 0.019 | 0.003 | 1.60E-09 |
| rs13016665 | 2 | 57995348 | C | A | 0.577 | -0.012 | 0.002 | 1.80E-09 |
| rs369230 | 16 | 89645437 | G | T | 0.308 | -0.013 | 0.002 | 1.80E-09 |
| rs10052591 | 5 | 50812738 | T | C | 0.573 | 0.012 | 0.002 | 2.10E-09 |
| rs3769949 | 2 | 1.66E+08 | T | A | 0.528 | -0.012 | 0.002 | 2.50E-09 |
| rs7155595 | 14 | 77502546 | A | C | 0.674 | -0.013 | 0.002 | 2.50E-09 |
| rs7077678 | 10 | 1.04E+08 | C | T | 0.623 | 0.012 | 0.002 | 2.60E-09 |
| rs860326 | 14 | 57342912 | C | T | 0.428 | 0.012 | 0.002 | 2.70E-09 |
| rs12202536 | 6 | 67475273 | A | G | 0.513 | -0.012 | 0.002 | 2.80E-09 |
| rs4814873 | 20 | 19616429 | C | T | 0.767 | 0.014 | 0.002 | 2.90E-09 |
| rs147412694 | 21 | 40702786 | G | A | 0.850 | -0.017 | 0.003 | 2.90E-09 |
| rs9842947 | 3 | 1.57E+08 | C | T | 0.326 | -0.013 | 0.002 | 3.10E-09 |
| rs2894808 | 6 | 52861990 | T | A | 0.922 | -0.022 | 0.004 | 3.50E-09 |
| rs12708665 | 16 | 24728227 | A | G | 0.285 | -0.013 | 0.002 | 3.50E-09 |
| rs202645 | 22 | 41798520 | A | G | 0.203 | -0.015 | 0.002 | 3.90E-09 |
| rs62098013 | 18 | 50863861 | G | A | 0.640 | -0.012 | 0.002 | 4.10E-09 |
| rs4957528 | 5 | 1.06E+08 | A | C | 0.208 | -0.015 | 0.002 | 4.20E-09 |
| rs1246265 | 9 | 86761745 | T | C | 0.305 | -0.013 | 0.002 | 4.20E-09 |
| rs6598539 | 15 | 99204483 | T | C | 0.489 | -0.012 | 0.002 | 4.50E-09 |
| rs13009008 | 2 | 1.74E+08 | A | G | 0.328 | 0.012 | 0.002 | 4.60E-09 |
| rs17553262 | 10 | 92912773 | A | C | 0.885 | -0.018 | 0.003 | 5.30E-09 |
| rs7297175 | 12 | 56473808 | T | C | 0.431 | -0.012 | 0.002 | 6.60E-09 |
| rs245774 | 5 | 1.71E+08 | A | G | 0.272 | -0.013 | 0.002 | 7.40E-09 |
| rs6962772 | 7 | 99081730 | A | G | 0.846 | 0.016 | 0.003 | 7.80E-09 |
| rs12481282 | 20 | 44761377 | G | C | 0.722 | -0.013 | 0.002 | 7.80E-09 |
| rs35343344 | 19 | 18471610 | C | A | 0.733 | 0.013 | 0.002 | 8.80E-09 |
| rs6562474 | 13 | 67332812 | C | G | 0.651 | 0.012 | 0.002 | 1.00E-08 |
| rs775758 | 3 | 77582005 | A | T | 0.433 | 0.012 | 0.002 | 1.10E-08 |
| rs2062882 | 8 | 91839576 | G | A | 0.587 | -0.012 | 0.002 | 1.10E-08 |
| rs7519626 | 1 | 99514554 | C | T | 0.324 | 0.012 | 0.002 | 1.20E-08 |
| rs9435340 | 1 | 1.08E+08 | T | A | 0.344 | 0.012 | 0.002 | 1.20E-08 |
| rs34866095 | 11 | 16377356 | A | G | 0.686 | -0.012 | 0.002 | 1.20E-08 |
| rs348809 | 20 | 59032097 | A | G | 0.348 | -0.012 | 0.002 | 1.30E-08 |
| rs1050847 | 16 | 87443734 | C | T | 0.426 | 0.011 | 0.002 | 1.40E-08 |
| rs73220544 | 3 | 1.31E+08 | A | C | 0.842 | -0.016 | 0.003 | 1.50E-08 |
| rs4571506 | 5 | 87756918 | C | T | 0.540 | 0.011 | 0.002 | 1.50E-08 |
| rs732083 | 17 | 37834367 | G | A | 0.333 | 0.012 | 0.002 | 1.50E-08 |
| rs6741228 | 2 | 22548774 | T | C | 0.433 | 0.011 | 0.002 | 1.60E-08 |
| rs4949465 | 1 | 32178489 | T | C | 0.870 | -0.017 | 0.003 | 1.70E-08 |
| rs62175972 | 2 | 1.61E+08 | T | C | 0.966 | 0.031 | 0.006 | 1.70E-08 |
| rs136233 | 22 | 31212410 | A | G | 0.809 | -0.014 | 0.003 | 1.80E-08 |
| rs12831617 | 12 | 84758368 | C | T | 0.764 | -0.013 | 0.002 | 1.90E-08 |
| rs11861214 | 16 | 746611 | G | T | 0.784 | 0.014 | 0.002 | 2.00E-08 |
| rs10918701 | 1 | 1.62E+08 | G | A | 0.372 | 0.012 | 0.002 | 2.10E-08 |
| rs10823968 | 10 | 74738269 | A | T | 0.633 | 0.012 | 0.002 | 2.10E-08 |
| rs74086911 | 12 | 50015942 | G | A | 0.925 | 0.021 | 0.004 | 2.10E-08 |
| rs4731925 | 7 | 1.33E+08 | C | T | 0.316 | -0.012 | 0.002 | 2.60E-08 |
| rs28485305 | 15 | 74044197 | C | T | 0.631 | 0.012 | 0.002 | 2.60E-08 |
| rs1193237 | 1 | 7526486 | G | C | 0.439 | -0.011 | 0.002 | 2.80E-08 |
| rs60952428 | 16 | 75640521 | T | C | 0.909 | 0.019 | 0.003 | 3.00E-08 |
| rs9904288 | 17 | 47031973 | T | C | 0.708 | 0.012 | 0.002 | 3.10E-08 |
| rs12967855 | 18 | 35138245 | A | G | 0.331 | 0.012 | 0.002 | 3.10E-08 |
| rs2254710 | 6 | 37477000 | C | A | 0.236 | 0.013 | 0.002 | 3.50E-08 |
| rs72674867 | 8 | 95578201 | A | T | 0.765 | 0.013 | 0.002 | 3.80E-08 |
| rs1931263 | 1 | 96175101 | G | T | 0.510 | -0.011 | 0.002 | 4.00E-08 |
| rs57611503 | 16 | 31165795 | G | A | 0.485 | 0.011 | 0.002 | 4.00E-08 |
| rs61796681 | 4 | 23678196 | A | T | 0.912 | -0.019 | 0.004 | 4.20E-08 |
| rs6957896 | 7 | 1.32E+08 | C | T | 0.503 | -0.011 | 0.002 | 4.50E-08 |
| rs2080870 | 5 | 60388313 | A | T | 0.258 | 0.012 | 0.002 | 4.90E-08 |

Note. CHR = chromosome, BP = base position, EA = effect allele, Non EA = non-effect allele, EAF = effect allele frequency.

**Table S2. Two-sample Mendelian randomisation analyses of the effect of lifetime smoking on coronary artery disease, lung cancer and *AHRR* Methylation.**

| **Outcome** | **Method** | **OR (95% CI)** | **P-value** |
| --- | --- | --- | --- |
| **Coronary Artery Disease** | Inverse-Variance Weighted | 1.56 (1.34, 1.82) | 1.19 × 10^-08^ |
|  | MR Egger (SIMEX) | 0.76 (0.44, 1.34) | 0.35 |
|  | Weighted Median | 1.65 (1.36, 2.00) | 5.40 × 10^-07^ |
|  | Weighted Mode | 1.79 (1.06, 3.03) | 0.03 |
|  | MR RAPS | 1.63 (1.40, 1.90) | 5.65 × 10^-10^ |
| **Lung Cancer** | Inverse-Variance Weighted | 4.21 (2.98, 5.96) | 3.49 × 10^-16^ |
|  | MR Egger (SIMEX) | 16.64 (3.88, 71.42) | 9.61 × 10^-05^ |
|  | Weighted Median | 2.77 (1.91, 4.03) | 8.88 × 10^-08^ |
|  | Weighted Mode | 6.19 (2.07, 18.54) | 0.001 |
|  | MR RAPS | 3.71 (2.75, 5.00) | 8.65 × 10^-18^ |
|  |  | **Beta (95% CI)** | **P-value** |
| ***AHRR* Methylation** | Inverse-Variance Weighted | -0.098 (-0.168, -0.028) | 0.006 |
|  | MR Egger (SIMEX) | -0.176 (-0.443, 0.102) | 0.217 |
|  | Weighted Median | -0.125 (-0.228, -0.021) | 0.02 |
|  | Weighted Mode | -0.207 (-0.511, 0.097) | 0.18 |
|  | MR RAPS | -0.095 (-0.171, -0.013) | 0.01 |

Of the 126 genome-wide significant SNPs associated with the lifetime smoking index, 126 were available from the GWAS of coronary artery disease (Nikpay et al., 2015), 120 from the GWAS of lung cancer (Wang et al., 2014) and 119 from our GWAS of *AHRR* locus methylation. SIMEX-corrected estimates are unweighted. Due to low regression dilution I^2^_GX_ (see Supplementary Table S3), MR Egger SIMEX estimates should be interpreted with caution and MR Egger estimates could not be calculated. SIMEX = simulation extrapolation, MR RAPS = robust adjusted profile score.

**Table S3. Tests of the unweighted and weighted regression dilution I^2^_GX._**

|  | I^2^_GX_ Unweighted | I^2^_GX_ Weighted | mF |
| --- | --- | --- | --- |
| Lifetime smoking > CAD | 0.644 | 0.454 | 44.05 |
| Lifetime smoking > Lung cancer | 0.643 | 0.340 | 44.36 |
| Lifetime smoking > AHRR methylation | 0.635 | 0.455 | 44.08 |
| Lifetime smoking > Schizophrenia (2014) | 0.634 | 0.405 | 44.08 |
| Lifetime smoking > Schizophrenia (Steiger filtered) | 0.667 | 0.441 | 44.25 |
| Lifetime smoking > Schizophrenia (2018) | 0.642 | 0.417 | 44.27 |
| Lifetime smoking > Schizophrenia (CHRNA5 removed) | 0.59 | 0.25 | 43.05 |
| Lifetime smoking > Depression (2018) | 0.644 | 0.429 | 44.05 |
| Lifetime smoking > Depression (2018) (Steiger filtered) | 0.646 | 0.436 | 44.22 |
| Lifetime smoking > Depression (2013) | 0 | 0 | 41.06 |
| Lifetime smoking > Depression (2019) | 0.617 | 0.363 | 43.46 |
| Schizophrenia (2014) > Lifetime smoking | 0.429 | 0 | 37.90 |
| Schizophrenia (2018) > Lifetime smoking | 0.487 | 0 | 40.86 |
| Schizophrenia (CHRNA3 removed) > Lifetime smoking | 0.43 | 0 | 37.78 |
| Depression (2018) > Lifetime smoking | 0 | 0 | 36.72 |
| Depression (2018) > Lifetime smoking (Steiger filtered) | 0 | 0 | 36.49 |
| Depression (2013) > Lifetime smoking | 0.358 | 0 | 19.05 |
| Depression (2019) > Lifetime smoking | 0.322 | 0.111 | 28.79 |
| Smoking initiation > Schizophrenia | 0.603 | 0.401 | 44.98 |
| Smoking initiation > Depression (2018) | 0.613 | 0.561 | 44.93 |
| Schizophrenia > Smoking initiation | 0.422 | 0.762 | 37.62 |
| Depression (2018) > Smoking initiation | 0 | 0 | 36.83 |

CAD: coronary artery disease. Unweighted estimates only take into account dilution in the SNP-exposure effects, whereas weighted estimates account for the SE of the SNP-outcome effects(Bowden, Del Greco M, et al., 2016). The unweighted I^2^ estimates were larger for both positive control outcomes so in Table 1 (main text), unweighted MR Egger SIMEX estimates are presented. For the main analysis unweighted MR Egger SIMEX corrections are presented unless I^2^ estimates < 0.6 which is too low to conduct either MR Egger analysis. All estimates show evidence of high dilution in the SNP-exposure effects, so MR Egger estimates should be interpreted with caution.

**Table S4. Tests of Heterogeneity in the SNP-exposure association**

|  | Method | Q | df | P-value |
| --- | --- | --- | --- | --- |
| Lifetime smoking > CAD | Inverse-variance weighted | 184.72 | 125 | <0.001 |
|  | MR Egger | 175.12 | 124 | 0.002 |
|  | Q’ | 9.60 | 1 | 0.002 |
| Lifetime smoking > Lung cancer | Inverse-variance weighted | 235.82 | 119 | <0.001 |
|  | MR Egger | 228.96 | 118 | <0.001 |
|  | Q’ | 6.87 | 1 | 0.009 |
| Lifetime smoking > AHRR methylation | Inverse-variance weighted | 122.07 | 118 | 0.38 |
|  | MR Egger | 121.35 | 117 | 0.37 |
|  | Q’ | 0.72 | 1 | 0.40 |
| Lifetime smoking > Schizophrenia | Inverse-variance weighted | 573.03 | 124 | <0.001 |
|  | MR Egger | 571.23 | 123 | <0.001 |
|  | Q’ | 1.81 | 1 | 0.18 |
| Lifetime smoking > Schizophrenia (2018) | Inverse-variance weighted | 592.94 | 121 | <0.001 |
|  | MR Egger | 591.77 | 120 | <0.001 |
|  | Q’ | 1.17 | 1 | 0.28 |
| Lifetime smoking > Schizophrenia (CHRNA5 variant removed) | Inverse-variance weighted | 566.23 | 123 | <0.001 |
|  | MR Egger | 565.96 | 122 | <0.001 |
|  | Q’ | 0.27 | 1 | 0.60 |
| Lifetime smoking > Depression (2018) | Inverse-variance weighted | 259.98 | 125 | <0.001 |
|  | MR Egger | 256.69 | 124 | <0.001 |
|  | Q’ | 3.29 | 1 | 0.07 |
| Lifetime smoking > Depression (2013) | Inverse-variance weighted | 33.014 | 33 | 0.46 |
|  | MR Egger | 33.07 | 32 | 0.41 |
|  | Q’ | 0.08 | 1 | 0.78 |
| Lifetime smoking > Depression (2019) | Inverse-variance weighted | 497.29 | 124 | <0.001 |
|  | MR Egger | 488.88 | 123 | <0.001 |
|  | Q’ | 8.41 | 1 | 0.003 |
| Schizophrenia > Lifetime smoking | Inverse-variance weighted | 773.92 | 101 | <0.001 |
|  | MR Egger | 770.30 | 100 | <0.001 |
|  | Q’ | 3.61 | 1 | 0.05 |
| Schizophrenia (2018) > Lifetime smoking | Inverse-variance weighted | 869.67 | 135 | <0.001 |
|  | MR Egger | 864.25 | 134 | <0.001 |
|  | Q’ | 5.42 | 1 | 0.20 |
| Schizophrenia (CHRNA3 variant removed) > Lifetime smoking | Inverse-variance weighted | 628.72 | 100 | <0.001 |
|  | MR Egger | 622.49 | 99 | <0.001 |
|  | Q’ | 6.23 | 1 | 0.01 |
| Depression (2018) > Lifetime smoking | Inverse-variance weighted | 255.61 | 33 | <0.001 |
|  | MR Egger | 250.29 | 32 | <0.001 |
|  | Q’ | 5.32 | 1 | 0.02 |
| Depression (2013) > Lifetime smoking | Inverse-variance weighted | 59.75 | 36 | 0.008 |
|  | MR Egger | 59.48 | 35 | 0.006 |
|  | Q’ | 0.27 | 1 | 0.60 |
| Depression (2019) > Lifetime smoking | Inverse-variance weighted | 481.97 | 94 | <0.001 |
|  | MR Egger | 481.77 | 93 | <0.001 |
|  | Q’ | 0.20 | 1 | 0.66 |
| Smoking Initiation > Schizophrenia | Inverse-variance weighted | 1468.61 | 370 | <0.001 |
|  | MR Egger | 1467.30 | 369 | <0.001 |
|  | Q’ | 1.32 | 1 | 0.25 |
| Smoking Initiation > Depression (2018) | Inverse-variance weighted | 704.94 | 369 | <0.001 |
|  | MR Egger | 699.86 | 368 | <0.001 |
|  | Q’ | 5.07 | 1 | 0.02 |
| Schizophrenia > Smoking initiation | Inverse-variance weighted | 409.42 | 106 | <0.001 |
|  | MR Egger | 402.35 | 105 | <0.001 |
|  | Q’ | 7.06 | 1 | <0.001 |
| Depression (2018) > Smoking initiation | Inverse-variance weighted | 153.61 | 33 | <0.001 |
|  | MR Egger | 150.95 | 32 | <0.001 |
|  | Q’ | 2.66 | 1 | 0.10 |

Note: df = degrees of freedom where degrees of freedom is equal to the number of SNPs -1. Q = Rucker’s Q(Bowden et al., 2017), a test of heterogeneity or dispersion in the SNP-exposure effects.

The Q’ indicates the extent to which MR Egger is a better fit than the inverse-variance weighted method.

**Table S5. MR Egger test of directional pleiotropy**

| **Outcome** | **Intercept (95% CI)** | **P-value** |
| --- | --- | --- |
| Lifetime smoking > CAD | 0.012 (0.003, 0.022) | 0.010 |
| Lifetime smoking > Lung cancer | -0.021 (-0.042, -0.001) | 0.062 |
| Lifetime smoking > AHRR methylation | -0.001 (-0.004, 0.002) | 0.406 |
| Lifetime smoking > Schizophrenia (2014) | -0.006 (-0.025, 0.013) | 0.531 |
| Lifetime smoking > Schizophrenia (2018) | -0.004 (-0.022, 0.013) | 0.627 |
| Lifetime smoking > Schizophrenia (CHRNA5 removed) | -0.002 (-0.022, 0.017) | 0.808 |
| Lifetime smoking > Depression (2018) | 0.006 (-0.003, 0.015) | 0.210 |
| Lifetime smoking > Depression (2013) | 0.009 (-0.055, 0.073) | 0.787 |
| Lifetime smoking > Depression (2019) | 0.005 (-0.002, 0.012) | 0.148 |
| Schizophrenia (2014) > Lifetime smoking | 0.002 (-0.004, 0.007) | 0.495 |
| Schizophrenia (2018) > Lifetime smoking | 0.002 (-0.002, 0.006) | 0.360 |
| Schizophrenia (CHRNA3 removed) > Lifetime smoking | 0.002 (-0.002, 0.007) | 0.32 |
| Depression (2018) > Lifetime smoking | 0.006 (-0.008, 0.019) | 0.416 |
| Depression (2013) > Lifetime smoking | -0.001 (-0.004, 0.003) | 0.693 |
| Depression (2019) > Lifetime smoking | 0.0004 (-0.004, 0.005) | 0.845 |
| Smoking initiation > Schizophrenia | -0.003 (-0.013, 0.007) | 0.565 |
| Smoking initiation > Depression (2018) | 0.004 (-0,001, 0.010) | 0.103 |
| Schizophrenia > Smoking initiation | 0.002 (-0.001, 0.006) | 0.177 |
| Depression (2018) > Smoking initiation | 0.003 (-0.012, 0.006) | 0.458 |

CAD: coronary artery disease.

**Table S6. Bi-directional two-sample Mendelian randomisation of lifetime smoking on schizophrenia and depression following Steiger filtering.**

| **Exposure** | **Outcome** | **Method** | **N SNP** | **OR (95% CI)** | **p-value** |
| --- | --- | --- | --- | --- | --- |
| **Smoking** | **Schizophrenia** | Inverse-Variance Weighted | 105/125 (84%) | 1.73 (1.39, 2.16) | 9.12 x 10^-07^ |
|  |  | MR Egger (SIMEX) |  | 6.19 (2.75, 13.95) | 2.64 x 10^-05^ |
|  |  | Weighted median |  | 1.72 (1.34, 2.22) | 2.78 x 10^-05^ |
|  |  | Weighted mode |  | 1.84 (0.73, 4.65) | 0.20 |
|  |  | MR RAPS |  | 1.83 (1.45, 2.32) | 3.97 x 10^-07^ |
| **Smoking** | **Depression** | Inverse-Variance Weighted | 124/126 (98%) | 1.94 (1.67, 2.25) | 3.46 x 10^-18^ |
|  |  | MR Egger (SIMEX) |  | 1.17 (0.67, 2.04) | 0.59 |
|  |  | Weighted median |  | 1.95 (1.65, 2.31) | 4.70 x 10^-15^ |
|  |  | Weighted mode |  | 1.81 (1.17, 2.79) | 0.008 |
|  |  | MR RAPS |  | 1.95 (1.68, 2.27) | 3.81 x 10^-18^ |
| **Exposure** | **Outcome** | **Method** | **N SNP** | **Beta (95% CI)** | **p-value** |
| **Schizophrenia** | **Smoking** | - | 102/102 (100%) | - | - |
| **Depression** | **Smoking** | Inverse-Variance Weighted | 32/34 (94%) | 0.063 (0.007, 0.120) | 0.028 |
|  |  | MR Egger (SIMEX) |  | - | - |
|  |  | Weighted median |  | 0.084 (0.042, 0.126) | 8.64 x 10^-05^ |
|  |  | Weighted mode |  | 0.111 (0.034, 0.187) | 0.008 |
|  |  | MR RAPS |  | 0.065 (0.008, 0.123) | 0.026 |

Note: For each SNP in the instrument, Steiger filtering calculates how much of the variance the SNP explains in the exposure and how much it explains in the outcome. The number of SNPs which explain more variance in the exposure are presented in the N SNP column. Analysis is then repeated using only these SNPs to ensure that results are not due to reverse causation. For the effect of schizophrenia on lifetime smoking, all SNPs comprising the instrument for schizophrenia were better instruments for schizophrenia than lifetime smoking, therefore, that analysis was not repeated. All SIMEX corrections are unweighted due to greater unweighted I^2^_GX_ (see Supplementary Table S2). Given low I^2^_GX_, all MR Egger results should be interpreted with caution.

**Table S7. Bi-directional two-sample Mendelian randomisation analyses of the effect of lifetime smoking on depression (2013).**

| **Exposure** | **Outcome** | **Method** | **OR (95% CI)** | **p-value** |
| --- | --- | --- | --- | --- |
| **Lifetime Smoking** | **Depression** | Inverse-Variance Weighted | 3.66 (2.08, 6.43) | 6.46 x 10^-06^ |
|  |  | Weighted median | 3.69 (1.72, 7.94) | 0.001 |
|  |  | Weighted mode | 3.18 (0.63, 16.06) | 0.17 |
|  |  | MR RAPS | 4.23 (2.33, 7.67) | 2.04 x 10^-06^ |
| **Exposure** | **Outcome** | **Method** | **Beta (95% CI)** | **p-value** |
| **Depression** | **Lifetime Smoking** | Inverse-Variance Weighted | 0.002 (-0.006, 0.011) | 0.572 |
|  |  | Weighted median | 0.003 (-0.007, 0.012) | 0.602 |
|  |  | Weighted mode | 0.002 (-0.016, 0.019) | 0.844 |
|  |  | MR RAPS | 0.003 (-0.005, 0.012) | 0.435 |

Note: When depression is the exposure, a relaxed p-value threshold of p<5x10^-5^ was used because there were no SNPs associated at the genome wide level of significance. The direction of effect is consistent with the more recent GWAS for MDD despite sample overlap. There is weaker statistical evidence, possibly due to reduced sample size (N = 18 759). Both MR Egger and MR Egger (SIMEX) estimates could not be conducted due to low regression dilution I^2^_GX_ (see Table S2).

**Table S8. Bidirectional two-sample Mendelian randomisation analyses of the effect of lifetime smoking (from a sensitivity GWAS without chip as a covariate) on schizophrenia and major depression.**

| **Exposure** | **Outcome** | **Method** | **N SNP** | **OR (95% CI)** | **P-value** |
| --- | --- | --- | --- | --- | --- |
| **Lifetime Smoking** | **Schizophrenia** | Inverse-Variance Weighted | 137 | 2.23 (1.69, 2.95) | 2.03 x 10^-08^ |
|  |  | MR Egger (SIMEX) | 137 | 3.71 (1.47, 3.39) | 0.006 |
|  |  | Weighted median | 137 | 2.07 (1.61, 2.66) | 1.24 x 10^-08^ |
|  |  | Weighted mode | 137 | 1.56 (0.63, 3.86) | 0.34 |
|  |  | MR RAPS | 137 | 2.42 (1.86, 3.15) | 6.09 x 10^-11^ |
| **Lifetime Smoking** | **Depression** | Inverse-Variance Weighted | 90 | 1.88 (1.57, 2.24) | 5.26 x 10^-12^ |
|  |  | MR Egger (SIMEX) | 90 | 4.39 (0.60, 323.15) | 0.51 |
|  |  | Weighted median | 90 | 1.70 (1.39, 2.07) | 1.53 x 10^-07^ |
|  |  | Weighted mode | 90 | 1.49 (0.99, 2.26) | 0.06 |
|  |  | MR RAPS | 90 | 1.85 (1.55, 2.21) | 9.00 x 10^-12^ |
|  |  |  |  | **Beta (95% CI)** | **P-value** |
| **Schizophrenia** | **Lifetime Smoking** | Inverse-Variance Weighted | 102 | 0.020 (0.003, 0.037) | 0.02 |
|  |  | MR Egger (SIMEX) | 102 | - | - |
|  |  | Weighted median | 102 | 0.008 (-0.004, 0.020) | 0.19 |
|  |  | Weighted Mode | 102 | 0.004 (-0.027, 0.035) | 0.80 |
|  |  | MR RAPS | 102 | 0.014 (-0.001, 0.030) | 0.06 |
| **Depression** | **Lifetime Smoking** | Inverse-Variance Weighted | 36 | 0.102 (0.036, 0.168) | 0.002 |
|  |  | MR Egger (SIMEX) | - | - | - |
|  |  | Weighted median | 36 | 0.101 (0.056, 0.147) | 1.29 x 10^-05^ |
|  |  | Weighted mode | 36 | 0.124 (0.044, 0.204) | 0.004 |
|  |  | MR RAPS | 36 | 0.091 (0.025, 0.157) | 0.007 |

Note: There were 139 genome-wide significant SNPs associated with lifetime smoking index when genotype chip was not included as a covariate in the GWAS. SIMEX-corrected estimates are unweighted. MR Egger regression was not conducted for schizophrenia or major depression as exposures because regression dilution I^2^_GX_ was below 0.3 (see Supplementary Table S6). Due to low regression dilution I^2^_GX_ for lifetime smoking as the exposure (see Supplementary Table S6), MR Egger and MR Egger SIMEX estimates should be interpreted with caution. SIMEX = simulation extrapolation, MR RAPS = robust adjusted profile score.

**Table S9. Bi-directional two-sample Mendelian randomisation analyses of the effect of lifetime smoking on schizophrenia (2018).**

| **Exposure** | **Outcome** | **Method** | **OR (95% CI)** | **p-value** |
| --- | --- | --- | --- | --- |
| **Lifetime Smoking** | **Schizophrenia** | Inverse-Variance Weighted | 2.64 (1.99, 3.52) | 2.36 x 10^-11^ |
|  |  | MR Egger (SIMEX) | 4.03 (1.38, 11.72) | 0.01 |
|  |  | Weighted median | 2.23 (1.76, 2.82) | 4.02 x 10^-11^ |
|  |  | Weighted mode | 2.20 (1.11, 4.36) | 0.02 |
|  |  | MR RAPS | 2.74 (2.09, 3.58) | 2.55 x 10^-13^ |
| **Exposure** | **Outcome** | **Method** | **Beta (95% CI)** | **p-value** |
| **Schizophrenia** | **Lifetime Smoking** | Inverse-Variance Weighted | 0.025 (0.011, 0.038) | 0.0003 |
|  |  | MR Egger (SIMEX) | - | - |
|  |  | Weighted median | 0.016 (0.005, 0.026) | 0.004 |
|  |  | Weighted mode | 0.029 (-0.011, 0.068) | 0.159 |
|  |  | MR RAPS | 0.017 (0.004, 0.03) | 0.009 |

Note: MR Egger estimates could not be conducted due to low regression dilution I^2^_GX_ (see Table S2).

**Table S10. Bi-directional two-sample Mendelian randomisation analyses of the effect of lifetime smoking on schizophrenia with the CHRNA5-A3-B4 Variants removed**

| **Exposure** | **Outcome** | **Method** | **N SNP** | **OR (95% CI)** | **p-value** |
| --- | --- | --- | --- | --- | --- |
| **Lifetime Smoking** | **Schizophrenia** | Inverse-Variance Weighted | 124 | 2.19 (1.61, 2.99) | 6.32 x 10^-07^ |
|  |  | Weighted median | 124 | 2.02 (1.57, 2.61) | 6.23 x 10^-08^ |
|  |  | Weighted mode | 124 | 1.67 (0.85, 3.28) | 0.14 |
|  |  | MR RAPS | 124 | 2.36 (1.77, 3.15) | 5.53 x 10^-09^ |
| **Exposure** | **Outcome** | **Method** |  | **Beta (95% CI)** | **p-value** |
| **Schizophrenia** | **Lifetime Smoking** | Inverse-Variance Weighted | 101 | 0.018 (0.003, 0.032) | 0.02 |
|  |  | Weighted median | 101 | 0.015 (0.003, 0.026) | 0.01 |
|  |  | Weighted mode | 101 | 0.016 (-0.012, 0.044) | 0.28 |
|  |  | MR RAPS | 101 | 0.016 (0.003, 0.030) | 0.02 |

Note: This analysis was run using Ripke et al (2014) GWAS of schizophrenia. When lifetime smoking was the exposure, rs8042849 (located in the CHRNA5 gene was removed). This variant is in high LD (r^2^ = 0.83) with rs16969968, the variant previously associated with smoking heaviness (Furberg et al., 2010). When schizophrenia was the exposure, we removed rs8042374. This is located in the gene CHRNA3, part of the CHRNA5-A3-B4 gene complex which has been shown to affect nicotine intake and consequently smoking heaviness (Furberg et al., 2010). However, this particular variant is in low LD with rs16969968 (r^2^ = 0.18). Results were highly consistent with those including these two variants. As the I^2^_GX_ statistic was low (indiciative of large regression dilution bias), MR Egger results were not reliable and so are not reported (see Table S2).

**Table S11. Bi-directional two-sample Mendelian randomisation analyses of the effect of lifetime smoking on depression (Howard et al., 2019 with UKBB and 23andMe removed)**

| **Exposure** | **Outcome** | **Method** | **N SNP** | **OR (95% CI)** | **p-value** |
| --- | --- | --- | --- | --- | --- |
| **Lifetime Smoking** | **Depression (2019)** | Inverse-Variance Weighted | 125 | 1.63 (1.46, 1.83) | 6.23 x 10^-17^ |
|  |  | MR Egger (SIMEX) | 125 | 0.89 (0.58, 1.37) | 0.59 |
|  |  | Weighted median | 125 | 1.59 (1.43, 1.75) | 3.06 x 10^-19^ |
|  |  | Weighted mode | 125 | 1.57 (1.15, 2.16) | 0.006 |
|  |  | MR RAPS | 125 | 1.61 (1.44, 1.81) | 9.14 x 10^-17^ |
| **Exposure** | **Outcome** | **Method** | **N SNP** | **Beta (95% CI)** | **p-value** |
| **Depression (2019)** | **Lifetime Smoking** | Inverse-Variance Weighted | 95 | 0.165 (0.126, 0.204) | 2.03 x 10^-16^ |
|  |  | MR Egger (SIMEX) | - | - | - |
|  |  | Weighted median | 95 | 0.131 (0.099, 0.164) | 1.69 x 10^-15^ |
|  |  | Weighted mode | 95 | 0.114 (0.035, 0.194) | 0.006 |
|  |  | MR RAPS | 95 | 0.171 (0.133, 0.201) | 7.13 x 10^-19^ |

Note: This analysis has been conducted using the most recent PGC GWAS of depression (Howard et al., 2019) with the UK Biobank and 23andMe samples removed. This is not the primary analysis given the less stringent definition of depression. However, the results are relatively consistent. Effects are smaller when depression is the outcome and slightly larger when depression is the exposure. SIMEX correction for lifetime smoking as the exposure was unweighted due to greater unweighted I^2^_GX_ (see Supplementary Table S2). Given low I^2^_GX_, all MR Egger SIMEX results should be interpreted with caution.

**Table S12. A comparison of each of the MR sensitivity analyses and their assumptions.**

| **Method** | **Description** | **Additional Assumptions** | **Power** | **Invalid variants allowed** | **IV2** | **IV3** |
| --- | --- | --- | --- | --- | --- | --- |
| Random effects Inverse‐variance weighted (IVW) | A meta-analysis of the Wald ratios for each SNP ($\frac{ZY}{ZX}$) weighted by the inverse of the variance of the SNP-outcome association. | Any horizontal pleiotropy must be balanced | Has the most power if the assumptions are satisfied. | 0% (or 100% if all horizontal pleiotropy is balanced) | ✗ | ✗ |
| MR‐Egger regression (Bowden et al., 2015) | An extension of the IVW which relaxes the assumption that any pleiotropy must be balanced. A significant intercept term suggests bias from directional pleiotropy, i.e. the average pleiotropic effect is not zero. MR-Egger regression provides consistent estimates even if all genetic instrumental variables are invalid as long as the INSIDE assumption is met. | InSIDE^a^  NOME^b^ | Has the lowest power | 100% | ✗ | ✓ |
| Weighted median (Bowden, Davey Smith, et al., 2016) | The weighted median estimate is obtained by first calculating the Wald ratio causal estimate for each SNP and then taking the estimate with the median inverse variance weight. | Consistent when 50% of weight contributed by genetic variants is valid. | Similar to that of IVW method. | 50% | ✓ | ✓ |
| Weighted mode (Hartwig et al., 2017) | Finds the largest cluster of Wald ratio estimates. The majority of the genetic instruments can be invalid providing the ZEMPA assumption is satisfied. In the weighted mode method, the mode is calculated using the inverse variance weights of the Wald ratios. | ZEMPA^c^ | Less powerful than IVW and weighted median. | 50% | ✓ | ✓ |
| Robust adjusted profile score (RAPS) (Zhao et al., 2018) | An extension of IVW into a general framework which allows very many weak instruments. Requires no sample overlap in the exposure and outcome SNP effect estimates. | InSIDE^a^  Pleiotropy is additive.  Pleiotropic effects are balanced (have mean zero). |  | 100% | ✓ | ✓ |

Note. Where Z = the genetic instrument, X = the exposure and Y = the outcome. IV2 = assumption 2, that all instruments (Z) must not be associated with confounders. IV3 = assumption 3, that all instruments (Z) must only be associated with the outcome (Y) through the exposure (X). These two columns have a cross if that method requires the assumption to be met and a tick if that assumption can be relaxed. Throughout the table, invalid refers to instruments that do not meet the required assumptions for MR. Power of the methods might differ under different models of pleiotropy. ^a^The InSIDE assumption = pleiotropic effects of Z are independent of the effects of Z on the exposure. ^b^The no measurement error assumption (NOME) = assumes that the ZX associations are known, rather than estimated. ^c^The ZEMPA assumption = the largest subset of genetic instrumental variables with the same ratio estimate will contain the valid instruments.

**References**

Bowden, J., Davey Smith, G., & Burgess, S. (2015). Mendelian randomization with invalid instruments: Effect estimation and bias detection through Egger regression. *International Journal of Epidemiology*, *44*(2), 512–525.

Bowden, J., Davey Smith, G., Haycock, P. C., & Burgess, S. (2016). Consistent estimation in Mendelian randomization with some invalid instruments using a weighted median estimator. *Genetic Epidemiology*, *40*(4), 304–314.

Bowden, J., Del Greco M, F., Minelli, C., Davey Smith, G., Sheehan, N. A., & Thompson, J. R. (2016). Assessing the suitability of summary data for two-sample Mendelian randomization analyses using MR-Egger regression: The role of the I 2 statistic. *International Journal of Epidemiology*, *45*(6), 1961–1974.

Bowden, J., Del Greco M, F., Minelli, C., Davey Smith, G., Sheehan, N., & Thompson, J. (2017). A framework for the investigation of pleiotropy in two-sample summary data Mendelian randomization. *Statistics in Medicine*, *36*(11), 1783–1802.

Boyd, A., Golding, J., Macleod, J., Lawlor, D. A., Fraser, A., Henderson, J., … Davey Smith, G. (2013). Cohort profile: The ‘children of the 90s’—the index offspring of the Avon Longitudinal Study of Parents and Children. *International Journal of Epidemiology*, *42*(1), 111–127.

Bulik-Sullivan, B. K., Loh, P.-R., Finucane, H. K., Ripke, S., Yang, J., Schizophrenia Working Group of the Psychiatric Genomics Consortium, … Neale, B. M. (2015). LD Score regression distinguishes confounding from polygenicity in genome-wide association studies. *Nature Genetics*, *47*(3), 291–295. https://doi.org/10.1038/ng.3211

Bycroft, C., Freeman, C., Petkova, D., Band, G., Elliott, L. T., Sharp, K., … Marchini, J. (2017). Genome-wide genetic data on ~500,000 UK Biobank participants. *BioRxiv*, 166298. https://doi.org/10.1101/166298

CARDIoGRAMplusC4D Consortium, & others. (2015). A comprehensive 1000 Genomes-based genome-wide association meta-analysis of coronary artery disease. *Nature Genetics*, *47*(10), 1121–1149.

Fraser, A., Macdonald-Wallis, C., Tilling, K., Boyd, A., Golding, J., Davey Smith, G., … others. (2012). Cohort profile: The Avon Longitudinal Study of Parents and Children: ALSPAC mothers cohort. *International Journal of Epidemiology*, *42*(1), 97–110.

Furberg, H., Kim, Y., Dackor, J., Boerwinkle, E., Franceschini, N., Ardissino, D., … Merlini, P. A. (2010). Genome-wide meta-analyses identify multiple loci associated with smoking behavior. *Nature Genetics*, *42*(5), 441.

Gaunt, T. R., Shihab, H. A., Hemani, G., Min, J. L., Woodward, G., Lyttleton, O., … Ho, K. (2016). Systematic identification of genetic influences on methylation across the human life course. *Genome Biology*, *17*(1), 61.

Hartwig, F. P., Smith, G. D., & Bowden, J. (2017). Robust inference in two-sample Mendelian randomisation via the zero modal pleiotropy assumption. *BioRxiv*, 126102.

Hemani, G., Zheng, J., Elsworth, B., Wade, K. H., Haberland, V., Baird, D., … Langdon, R. (2018). The MR-Base platform supports systematic causal inference across the human phenome. *ELife*, *7*, e34408.

Houseman, E. A., Accomando, W. P., Koestler, D. C., Christensen, B. C., Marsit, C. J., Nelson, H. H., … Kelsey, K. T. (2012). DNA methylation arrays as surrogate measures of cell mixture distribution. *BMC Bioinformatics*, *13*(1), 86.

Howard, D. M., Adams, M. J., Clarke, T.-K., Hafferty, J. D., Gibson, J., Shirali, M., … McIntosh, A. M. (2019). Genome-wide meta-analysis of depression identifies 102 independent variants and highlights the importance of the prefrontal brain regions. *Nature Neuroscience*, *22*(3), 343. https://doi.org/10.1038/s41593-018-0326-7

Lawlor, D. A., Tilling, K., & Davey Smith, G. (2016). Triangulation in aetiological epidemiology. *International Journal of Epidemiology*, *45*(6), 1866–1886.

Leffondré, K., Abrahamowicz, M., Siemiatycki, J., & Rachet, B. (2002). Modeling smoking history: A comparison of different approaches. *American Journal of Epidemiology*, *156*(9), 813–823.

Lewer, D., McKee, M., Gasparrini, A., Reeves, A., & de Oliveira, C. (2017). Socioeconomic position and mortality risk of smoking: Evidence from the English Longitudinal Study of Ageing (ELSA). *The European Journal of Public Health*, *27*(6), 1068–1073.

Liu, M., Jiang, Y., Wedow, R., Li, Y., Brazel, D. M., Chen, F., … Tian, C. (2019). Association studies of up to 1.2 million individuals yield new insights into the genetic etiology of tobacco and alcohol use. *Nature Genetics*, 1.

Mitchell, R., Hemani, G., Dudding, T., & Paternoster, L. (2017, November 6). UK Biobank Genetic Data: MRC-IEU Quality Control, Version 1. https://doi.org/10.5523/bris.3074krb6t2frj29yh2b03x3wxj

Munafò, M. R., Timofeeva, M. N., Morris, R. W., Prieto-Merino, D., Sattar, N., Brennan, P., … Davey Smith, G. (2012). Association Between Genetic Variants on Chromosome 15q25 Locus and Objective Measures of Tobacco Exposure. *JNCI: Journal of the National Cancer Institute*, *104*(10), 740–748. https://doi.org/10.1093/jnci/djs191

Pidsley, R., Wong, C. C., Volta, M., Lunnon, K., Mill, J., & Schalkwyk, L. C. (2013). A data-driven approach to preprocessing Illumina 450K methylation array data. *BMC Genomics*, *14*(1), 293.

Purcell, S., Neale, B., Todd-Brown, K., Thomas, L., Ferreira, M. A., Bender, D., … others. (2007). PLINK: A tool set for whole-genome association and population-based linkage analyses. *The American Journal of Human Genetics*, *81*(3), 559–575.

R. Core Team. (2014). *R: A language and environment for statistical computing. R Foundation for Statistical Computing, Vienna, Austria. 2013*. ISBN 3-900051-07-0.

Relton, C. L., Gaunt, T., McArdle, W., Ho, K., Duggirala, A., Shihab, H., … Reik, W. (2015). Data resource profile: Accessible resource for integrated epigenomic studies (ARIES). *International Journal of Epidemiology*, *44*(4), 1181–1190.

Ripke, S., Neale, B. M., Corvin, A., Walters, J. T., Farh, K.-H., Holmans, P. A., … others. (2014). Biological insights from 108 schizophrenia-associated genetic loci. *Nature*, *511*(7510), 421–427.

Thorgeirsson, T. E., Geller, F., Sulem, P., Rafnar, T., Wiste, A., Magnusson, K. P., … Stefansson, K. (2008). A Variant Associated with Nicotine Dependence, Lung Cancer and Peripheral Arterial Disease. *Nature*, *452*(7187), 638–642. https://doi.org/10.1038/nature06846

Tobacco Consortium. (2010). Genome-wide meta-analyses identify multiple loci associated with smoking behavior. *Nature Genetics*, *42*(5), 441–447.

Touleimat, N., & Tost, J. (2012). Complete pipeline for Infinium® Human Methylation 450K BeadChip data processing using subset quantile normalization for accurate DNA methylation estimation. *Epigenomics*, *4*(3), 325–341.

Wang, Y., McKay, J. D., Rafnar, T., Wang, Z., Timofeeva, M. N., Broderick, P., … Han, Y. (2014). Rare variants of large effect in BRCA2 and CHEK2 affect risk of lung cancer. *Nature Genetics*, *46*(7), 736.

Ware, J. J., van den Bree, M. B., & Munafò, M. R. (2011). Association of the CHRNA5-A3-B4 gene cluster with heaviness of smoking: A meta-analysis. *Nicotine & Tobacco Research*, *13*(12), 1167–1175.

Wray, N. R., Sullivan, P. F., & others. (2018). Genome-wide association analyses identify 44 risk variants and refine the genetic architecture of major depression. *Nature Genetics*, *50*, 668–681.

Zhao, Q., Wang, J., Hemani, G., Bowden, J., & Small, D. S. (2018). Statistical inference in two-sample summary-data Mendelian randomization using robust adjusted profile score. *ArXiv*. Retrieved from http://arxiv.org/abs/1801.09652
